# Timing of Allocentric and Egocentric Spatial Processing in Human Intracranial EEG

**DOI:** 10.1007/s10548-023-00989-2

**Published:** 2023-07-21

**Authors:** Sofiia Moraresku, Jiri Hammer, Radek Janca, Petr Jezdik, Adam Kalina, Petr Marusic, Kamil Vlcek

**Affiliations:** 1 Laboratory of Neurophysiology of Memory, Institute of Physiology, Czech Academy of Sciences, Videnska 1083, 142 20, Prague, Czechia; 2 Third Faculty of Medicine, Charles University, Prague, Czechia; 3 Department of Neurology, Second Faculty of Medicine, Charles University and Motol University Hospital, Prague, Czechia; 4 Department of Circuit Theory, Faculty of Electrical Engineering, Czech Technical University in Prague, Prague, Czechia

**Keywords:** Intracranial EEG, High-frequency gamma activity, Reference frames, Allocentric, Egocentric, Spatial judgment

## Abstract

Spatial reference frames (RFs) play a key role in spatial cognition, especially in perception, spatial memory, and navigation. There are two main types of RFs: egocentric (self-centered) and allocentric (object-centered). Although many fMRI studies examined the neural correlates of egocentric and allocentric RFs, they could not sample the fast temporal dynamics of the underlying cognitive processes. Therefore, the interaction and timing between these two RFs remain unclear. Taking advantage of the high temporal resolution of intracranial EEG (iEEG), we aimed to determine the timing of egocentric and allocentric information processing and describe the brain areas involved. We recorded iEEG and analyzed broad gamma activity (50–150 Hz) in 37 epilepsy patients performing a spatial judgment task in a three-dimensional circular virtual arena. We found overlapping activation for egocentric and allocentric RFs in many brain regions, with several additional egocentric- and allocentric-selective areas. In contrast to the egocentric responses, the allocentric responses peaked later than the control ones in frontal regions with overlapping selectivity. Also, across several egocentric or allocentric selective areas, the egocentric selectivity appeared earlier than the allocentric one. We identified the maximum number of egocentric-selective channels in the medial occipito-temporal region and allocentric-selective channels around the intraparietal sulcus in the parietal cortex. Our findings favor the hypothesis that egocentric spatial coding is a more primary process, and allocentric representations may be derived from egocentric ones. They also broaden the dominant view of the dorsal and ventral streams supporting egocentric and allocentric space coding, respectively.

**Supplementary Information:**

The online version contains supplementary material available at 10.1007/s10548-023-00989-2.

## Introduction

Spatial reference frames (RFs) shape our understanding of many cognitive processes involved in spatial cognition, such as perception, performing actions in space, and navigation. RFs also play a crucial role in spatial memory allowing information storage to be organized into various coordinate systems. Broadly speaking, in the egocentric RF, the locations of objects are encoded with respect to the position and heading of the subject, while in the allocentric RF, they are encoded relative to each other or to environmental landmarks and do not depend on the position of the subject (Klatzky 1998).

It has been suggested that separate neural circuits support these two types of spatial coding. The well-known perception-action model (Goodale and Milner 1992; Goodale et al. 2004) implicates that the dorsal (in the parietal cortex) and ventral (in the temporal cortex) streams process visual information for different purposes, i.e., for motor action and conscious perception, respectively. According to this model, the dorsal stream needs to compute the exact position of the target relative to the subject to perform accurate goal-directed actions in real-time. For example, to grasp a cup of coffee successfully, one needs to know its exact position relative to themselves. However, the dorsal stream is not unitary; it seems to consist of three sub-pathways with distinct functions (Kravitz et al. 2011). The parieto-prefrontal, parieto-premotor, and parieto-medial temporal pathways presumably support both conscious and non-conscious visuospatial processing, including spatial working memory, visually guided action, and navigation, respectively. In contrast, the ventral stream computes the size, location, or orientation of an object primarily with respect to other objects or landmarks in the environment to perceive or remember that object. Therefore, this model associates egocentric and allocentric coding with the dorsal and ventral visual streams, respectively. Although recent studies suggest the two streams are interconnected and more integrated (Hutchison and Gallivan 2018; Ray et al. 2020), the perception-action model still provides a useful framework for understanding the visuospatial functions.

The results of many studies have shown evidence supporting this association between two visual streams and two spatial RFs. A series of fMRI studies investigated neural correlates of allocentric and egocentric RFs during spatial judgment and navigation tasks and showed the involvement of separate brain areas for two types of spatial coding. Specifically, egocentric RF use was accompanied by dominant activity in the superior parietal lobule, precuneus, superior, middle and inferior frontal gyri (Committeri et al. 2004; Galati et al. 2000; Parslow et al. 2004; Rosenbaum et al. 2004; Ruotolo et al. 2019; Saj et al. 2014), while allocentric RF use was supported by activation in the lateral and ventromedial occipito-temporal cortex (Committeri et al. 2004; Galati et al. 2000; Ruotolo et al. 2019; Saj et al. 2014), and also in the hippocampus in spatial navigation studies (Hirshhorn et al. 2012; Iaria et al. 2007; Jordan et al. 2004; Maguire et al. 1998; Rodriguez 2010; Spiers and Maguire 2007; see also review Moraresku and Vlcek 2020). Egocentric and allocentric coding are tightly connected with spatial attentional control, which in the healthy brain has been linked to the activation of a distributed frontoparietal attention network (Corbetta and Shulman 2002; Szczepanski et al. 2010). The influence of spatial attention control on the two types of spatial processing has been shown in studies of brain-damaged patients suffering from hemispatial neglect. Hemispatial neglect is characterized by the inability to direct attention to the contralateral visual field. Lesions only to the fronto-parietal areas were more often associated with its egocentric form, i.e. the inability of patients to perceive space on the contralesional side of their body, while lesions including the occipito-temporal areas were related to the allocentric neglect, i.e. inability of patients to perceive the contralesional side of individual objects, independently of their own position (Chechlacz et al. 2012; Grimsen et al. 2008).

However, a few fMRI studies found no clear distinction between dorsal and ventral stream activity for egocentric and allocentric processing, respectively. In an experimental paradigm with only a verbal description of spatial relations and without the visual presentation of the task, Zaehle et al. (2007) found that inferior and superior parietal lobules (dorsal areas) were more active in the allocentric task compared to the egocentric one. The greater allocentric activation in the parietal cortex may be associated with additional efforts to mentally translate object-relative (i.e. allocentric) spatial locations into new egocentric positions, needed for the behavioral response (Filimon 2015). Weniger et al. (2010) studied spatial navigation in a virtual maze without any landmarks, whereby forcing participants to use an egocentric strategy, and found activation in the parahippocampal and lingual gyri (i.e., in the ventral stream). Other studies linked the hippocampal activation with egocentric-updating processes (Gomez et al. 2012, 2014). For instance, a patient with bilateral hippocampal damage had difficulties with tasks requiring processing and integration of egocentric self-motion information, while his performance in allocentric tasks was comparable to the control group (Gomez et al. 2012). Also, a recent meta-analysis (Li et al. 2021) examining neural representations of RFs during spatial navigation in humans found a stronger activation for the allocentric RF in the middle frontal gyrus and cerebellar culmen, and common clusters of activation in the parahippocampal and lingual gyri, as well as the precuneus. In addition, according to another recent meta-analysis, activity associated with the allocentric and egocentric RFs across various experimental paradigms converges in the right parietal and the right frontal cortex (Derbie et al. 2021a). Therefore, neural processes underlying egocentric and allocentric RFs seem to be at least partially overlapping, with both visual streams and frontal cortex engaged in two RFs. The distinction between neural circuits underlying egocentric and allocentric RFs and their localization in the dorsal and ventral streams is thus still inconclusive.

Moreover, egocentric and allocentric RFs may also differ in the temporal profile of neural processing. Almost all the previous studies about egocentric and allocentric RFs used functional neuroimaging methods with an inherently slow temporal resolution, which mostly showed only the involvement of specific brain areas but not the temporal dynamics of the underlying cognitive processes. To date, only one intracranial EEG (iEEG) study has investigated the encoding of RFs in iEEG, concentrating mostly on the parahippocampal place area (PPA). The authors described several scene processing stages, with a common phase for allocentric and egocentric processing at 400–600 ms after stimulus onset, followed by a specific allocentric processing stage at 600–800 ms (Bastin et al. 2013). Still, it is unclear whether other brain areas involved in spatial processing, especially those with overlapping activity for egocentric and allocentric RFs, share timely separated processes as in PPA.

In the current study, taking advantage of the high temporal resolution of iEEG, we aimed to disentangle the timing of egocentric and allocentric information processing across brain regions involved in spatial perception and ascertain whether they show dissociated timing patterns for egocentric and allocentric RFs analogous to PPA. Thus, our objective was to broaden the results of Bastin et al. (2013) study on other brain regions. We employed a spatial distance estimation task, similar to previous studies (Committeri et al. 2004; Bastin et al. 2013), but here using a three-dimensional (3D) circular virtual arena. The task for the subjects was to estimate which of the two objects on the floor was closer (i) to a landmark at the wall, assuming allocentric RF, or (ii) to the subject, assuming egocentric RF. In our analysis, we focused on broadband gamma activity (BGA, 50–150 Hz) as it has a strong positive correlation with the fMRI blood-oxygen-level-dependent (BOLD) signal (Mukamel et al. 2005) and local neuronal firing rate (Manning et al. 2009), and has also been used as a general index of cortical processing (Lachaux et al. 2012) in many cognitive and motor tasks (Bastin et al. 2013; Musch et al. 2014; Hammer et al. 2016; Vlcek et al. 2020). Assuming partially overlapping neural processes underlying egocentric and allocentric RFs, we expected to find BGA responses evoked by both egocentric and allocentric RFs in frontal, temporal and parietal areas, with temporally separated processes for egocentric and allocentric RFs, similarly to scene processing stages found in PPA (Bastin et al. 2013). More specifically, in this fronto-temporo-parietal network, we expected to observe a delayed activity for the allocentric RF compared to the egocentric RF, based on the premise that, during scene visual processing, allocentric representations are derived via mental transformations of primary egocentric ones (Byrne et al. 2007; Filimon 2015). Similarly, we expected to find the earlier egocentric selectivity in the egocentric-selective regions than the allocentric selectivity in allocentric-selective brain regions.

## Materials and Methods

### Stimuli and Task

**Fig. 1:**
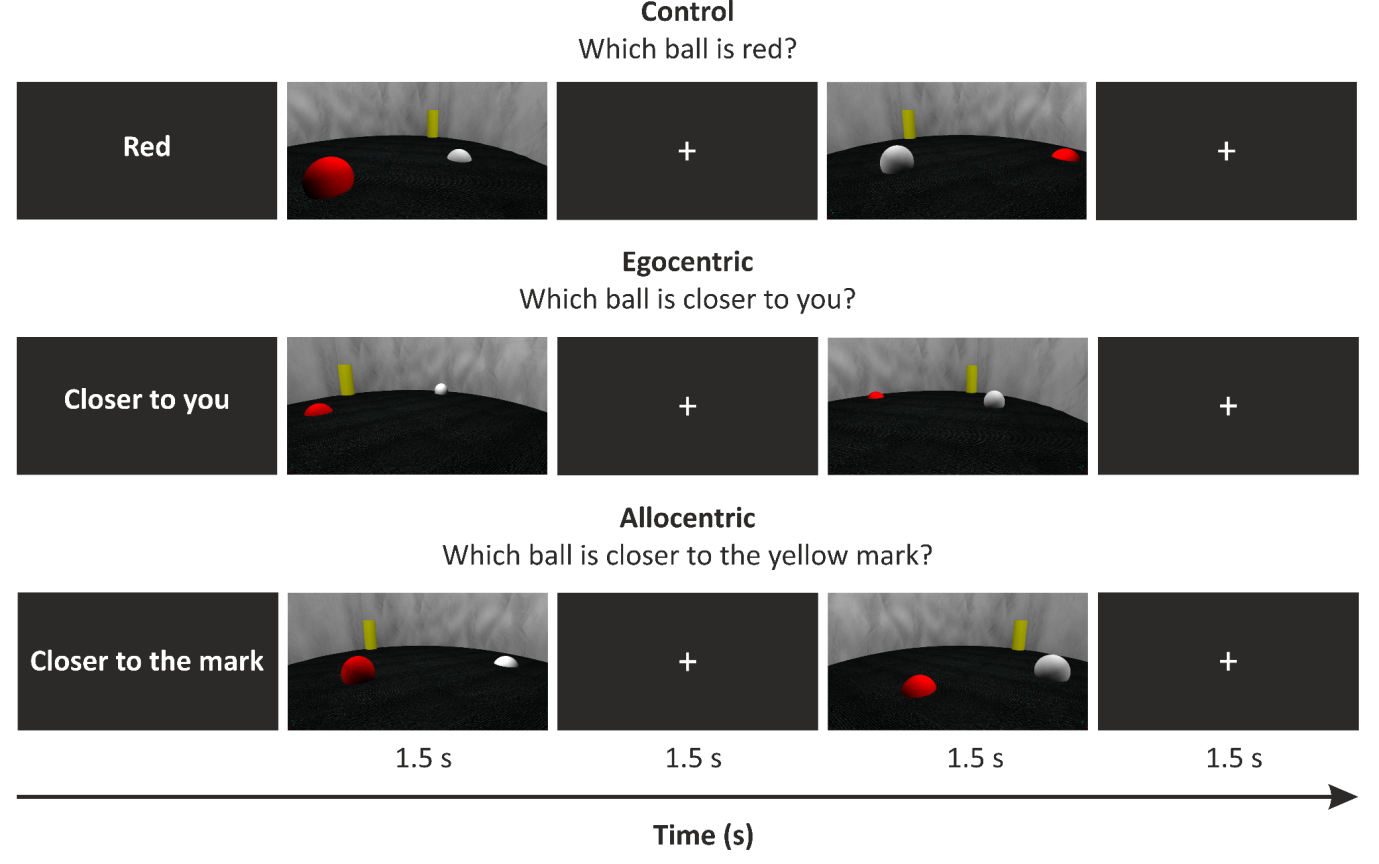
The experimental design of the task. Stimuli were delivered in blocks; each block was assigned to one particular condition: control, egocentric, or allocentric. The task was to judge which ball was closer to the current point of view of the participant (egocentric) or closer to the yellow mark (allocentric). In the control condition, participants were required to choose which ball was red. The subjects pressed either a left or right arrow on the keyboard to indicate the ball of their choice. The lower timeline shows the timing of each trial

We used an Unreal Editor (UT 2004 EpicGames, 2004) to create 3D scenes of the virtual environment of a circular arena (imitating Morris water maze, see e.g., Fajnerova et al. 2014) containing three objects: a yellow mark located at the wall and red and white balls (see Fig. 1). A total of 128 unique images of 3D scenes were produced with variable mark and ball positions and a variable point of view. During the experiment, patients were asked to judge which ball was closer to their current point of view (egocentric condition) or closer to the yellow mark (allocentric condition). In addition, we employed a control condition with similar low-level visual, attentional, and motor components, irrelevant for the reference frame use, whereby patients were asked to judge which ball was red. Each image was used three times, i.e., under egocentric, allocentric, and control conditions, respectively. To prevent the subjects from using alternative non-spatial strategies (e.g., making a choice based on apparent object-size characteristics or two-dimensional (2D) on-screen distances), we created several types of images that differed in terms of the strategies patients may potentially employ instead of spatial estimates. For example, in half of the images, egocentric and allocentric distance estimation was congruent: the same ball was closer to the patients and the yellow mark. In contrast, in the other half of the images, egocentric and allocentric distance estimation did not correspond: the ball that was closer to the subject was further from the yellow mark (supplementary Fig. S1A). In some images, allocentric estimation was the same in three-dimensional space and the two-dimensional coordinates of the screen (Allo 3D = 2D), while in others, it differed - the ball that was closer to the yellow mark in three-dimensional space was further in two-dimensional coordinates of the screen (Allo 3D ≠ 2D, supplementary Fig. S1B, Fig. S2). Furthermore, the images differed in the relative size of the correct ball within the egocentric coordinates - in some images, the ball that was closer to the subject was larger, but in others, it was smaller (supplementary Fig. S1C). Similarly, the images differed in terms of the allocentric coordinates - the smaller or larger ball was closer to the yellow mark (supplementary Fig. S1D). We expected the estimates using a three-dimensional mental model of the scene to be the only consistently successful strategy in such an experimental design.

The experiment lasted approximately 30 min and consisted of 384 test trials (128 per condition) divided into eight sessions. Each session included three blocks consisting of 16 trials. Each block was assigned to one particular condition (control, egocentric, or allocentric), but the order of blocks was counterbalanced, with a pause between them of a subject-controlled length. At the beginning of each block, the patients received simple on-screen instructions about the condition in the upcoming block. The patients were required to press a key to start the block; after which a series of sixteen 3 s trials followed. Each trial included the presentation of a three-dimensional scene for 1.5 s, followed by the presentation of a white fixation cross for 1.5 s. The patients answered the question using the arrows on a keyboard: left ball = left arrow, right ball = right arrow. At the end of each block, the patients were given feedback on their performance including the number of correct responses and their average reaction time to motivate them to perform the task correctly. The test trials were preceded by a training session with shortened blocks consisting of five trials per condition and feedback for the patients after each trial. Because of the large interindividual variability between the patients during the training session, these data were not included in the analysis and were not counted in the 384 test trials.

Visual stimuli were delivered using the PsychoPy 1.84 environment (Peirce et al. 2019) on a 15.6-inch TFT notebook monitor with a refresh rate of 60 Hz. The monitor was positioned approximately 60 cm from the subject’s eyes, making the stimuli cover 10° of the visual field. The stimulus presentation and the EEG recording were synchronized using TTL pulses sent to an EEG acquisition PC with each stimulus.

### Patients

A total of 37 patients (23 women; from 19 to 54 years old, median age 32 years old; education level: four primary school, 24 secondary school, and nine college) with drug-resistant epilepsy participated in our study from the Motol Epilepsy Center in Prague. The patients underwent intracranial EEG (iEEG) monitoring for precise localization of the epileptic seizure onset zone before surgery. All the patients signed an informed consent to participate and the study was approved by the Ethics Committee of Motol University Hospital. All the patients had normal or corrected to normal vision.

### Electrode Implantation and Intracranial EEG Recordings

IEEG was recorded with stereotactically implanted multi-contact electrodes, often also referred to as stereo-EEG (sEEG). Recording sites were selected on an individual basis, strictly according to the medical requirements of the presurgical evaluation of epileptic zones, with no reference to the present study. Eleven to 15 semi-rigid electrodes were implanted per patient intracerebrally, depending on the suspected origin of their seizures. Each electrode had a diameter of 0.8 mm and consisted of eight to 18 contacts of 2 mm in length, 1.5 mm apart (DIXI Medical Instruments). Postimplantation CT coregistered to preimplantation MRI was used to identify the positions of electrode contacts in each patient. The anatomical positions of the electrode contacts were visually verified by an experienced neurologist. The contact positions were normalized to the Montreal Neurological Institute (MNI) space using standard Statistical Parametric Mapping algorithms (SPM 12). All coordinates (x, y, z) are given in MNI space. The iEEG signal was recorded using two different video-EEG monitoring systems: Natus NicoleteOne (in 22 patients) or Natus Quantum. The data were sampled at 512, 2048, or 8000 Hz, depending on the amplifier, using a reference electrode located in the white matter.

**Fig. 2:**
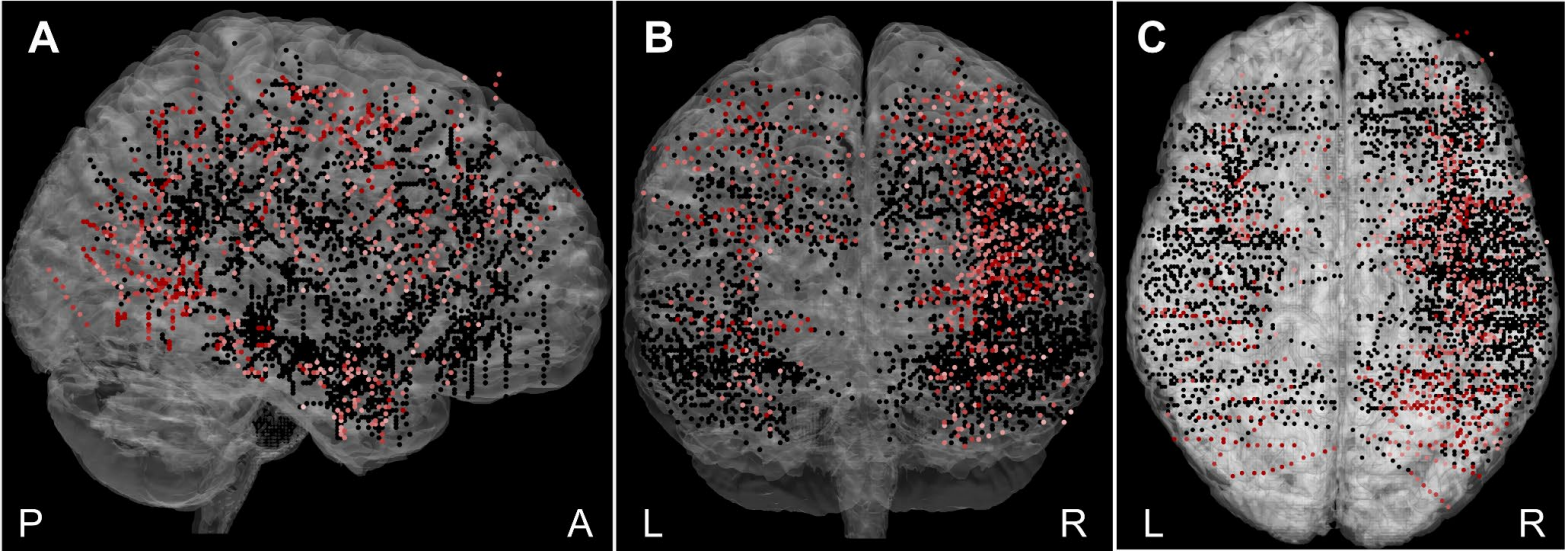
The plot of all 4586 recorded channels (including also heterotopic cortex channels with inaccurate MNI coordinates, excluded from all analyses) across 37 patients on a standard MNI brain in the **(A)** sagittal, **(B)** coronal, and **(C)** axial plane. In total, 546 active channels showing a significant response relative to the baseline to at least one condition - control, egocentric, or allocentric - are plotted in shades of red (the darker shade represents the higher response magnitude); non-responding channels are plotted in black. P, posterior; A, anterior; L, left; and R, right

### iEEG Analysis

We used a custom package developed in our laboratory (freely available at https://github.com/kamilvlcek/iEEG_scripts/releases/tag/v2.0.0) in MATLAB 9.4 (Mathworks, Inc.) to perform the time-frequency analysis of iEEG data (Vlcek et al. 2020). First, we resampled all the data to 512 Hz and excluded the electrode contacts with obvious artifacts from further analysis. From the entire iEEG recording, we computed bipolar derivations between adjacent contacts to suppress contributions from distant neuronal assemblies and considered bipolar iEEG signals originating from a cortical volume centered between two contacts. Here, we refer to one bipolar contact pair as a ‘channel’. When the channel was derived from two contacts in a different brain structure, we labeled it with the structure with a larger unilateral response. In total, iEEG activity was recorded from 4586 bipolar channels (see Fig. 2) from 37 patients, with the prevalent number of recording sites in the right hemisphere (3302, 72%).

We focused on the analysis of broadband gamma activity (BGA, 50–150 Hz) as it has a strong positive correlation with the fMRI BOLD signal (Mukamel et al. 2005; Ojemann et al. 2010) and local neuronal firing rate (Manning et al. 2009). Instantaneous amplitude was estimated using the following procedure (the same as in Vlcek et al. 2020): the entire recording dataset was band-pass filtered in consecutive non-overlapping 5 Hz frequency bands in the broad gamma range (e.g., 50–55, 55–60, …, 145–150 Hz). For each frequency band, the amplitude envelope was extracted using a Hilbert transform; the obtained envelope was downsampled to 64 Hz, resulting in a time resolution of 15.625 ms. Subsequently, the envelope of each band was divided by its mean value over the entire recording session, effectively whitening the broad frequency band and compensating for the 1/f frequency decay of EEG signals (Miller et al. 2014). All the bands were then averaged together and multiplied by 100 to obtain a single time series of BGA power for each channel expressed in the percentages of the mean value, and this signal was divided into epochs of between -500 and 1500 ms relative to the stimulus onset. The mean of the prestimulus interval (-50 to 0 ms) was subtracted from each epoch to remove signal changes independent of the respective stimulus. We excluded epochs in each channel containing interictal epileptiform discharges, which were identified by a spike detector implemented in MATLAB (https://github.com/EpiReC-ISARG/IED_detector, Janca et al. 2015) from further analysis. Also, from the iEEG analysis, we excluded trials (median 38, range 3-191 of all 384 trials across the 37 patients) with an incorrect or too slow (i.e. not within 3s after the stimulus) behavioral response and the blocks of trials if the mean accuracy of that block was below 75%, implying that the patient did not understand the instruction of the block and responded close to the chance level.

We used BGA responses to identify ‘active’ channels showing a significant response for at least one condition compared to the baseline. For each channel, we compared the average BGA for all trials of the respective condition during the pre-stimulus interval (-500–0 ms) with all the time points during the post-stimulus period (0–1500 ms) using a Wilcoxon signed-rank test corrected for multiple comparisons across the time samples (because of the non-normal data distribution and similar to Bastin et al. 2013; Musch et al. 2014; Vlcek et al. 2020) with a false discovery rate (FDR) procedure (Genovese et al. 2002). As a conservative estimate, we used a sliding window of six samples (93.75 ms) with the highest p-value. If there was a significant difference at any time point relative to the baseline for a selected condition, the channel was considered active. We found 801 such channels. Of these, we excluded 255 channels localized in the white matter or heterotopic cortex and those showing very late response connected to the key press (i.e., when the BGA peak was more than 800 ms after the stimulus onset, and on the plot of all individual epochs appeared aligned to the key press time) or containing obvious artifacts. The remaining 546 channels comprised the pool of active channels (see Fig. 2 for their positions in the brain).

Then, we performed three types of analyses of the BGA responses. Firstly, we directly compared BGA responses between conditions in each active channel separately. We used a Wilcoxon signed-rank test with FDR correction across the time samples and all active channels to compare BGA response in the post-stimulus period (0-1500 ms) for all individual time points between any two conditions (allocentric vs. egocentric, egocentric vs. allocentric, allocentric vs. control, egocentric vs. control). We labeled two types of channels ‘allocentric-selective’, i.e., channels showing a significantly higher response for allocentric than for egocentric condition and channels showing a significantly higher response for allocentric than for control condition but at the same time, without a significant difference between egocentric and control conditions (see Fig. 4B). The same principle was behind labeling ‘egocentric-selective’ channels (see Fig. 4A). The third category, labeled ‘spatial-selective’, consisted of channels in which both allocentric and egocentric responses were significantly higher than the control (see Fig. 4C).

We grouped these three categories of channels based on their anatomical locations (neurology labels from a neurologist according to Mai et al. 2015 and MNI coordinates) into nine brain regions (regions of interest, ROIs, listed with details in the Results section). To ensure the inter-subject reproducibility of our results, we further focused only on areas that included channels from at least three different patients (Lachaux et al. 2012). This left us with a set of channels of interest (ChOIs), which were used in the following two analyses. For these nine ROIs, we applied the χ² test to check whether the proportion of egocentric-selective, allocentric-selective, and spatial-selective channels was the same in each brain region.

Using the ChOIs, we performed a second analysis to characterize the responses to each condition across the ROIs, independent of the channel selectivity. To specify the time course of responses, we applied two measures of temporal dynamics based on our BGA sampling frequency (64 Hz): onset latency - the first time bin at which a significant *p*-value was observed relative to the baseline (tsig), and peak latency - the time when the power change of response reached 90% of its maximum relative to the baseline for the first time (t90). Also, we compared the magnitude of the response - the maximal increase of BGA in the percentage of baseline activity - across brain regions. To compare all these measures, we used two-way mixed ANOVA (similar to Bastin et al. 2013; Musch et al. 2014; Vlcek et al. 2020) with the within-subject factor **Condition** and between-subject factor **ROI** with a post hoc Tukey HSD test to correct for multiple comparisons (Abdi & Williams 2010) with a significance level of *p* < 0.05.

In the third analysis, also using the ChOIs, we more accurately characterized the temporal dynamics associated with egocentric and allocentric coding during the post-stimulus period. To avoid the jitter in the BGA temporal profile and arrive at a statistically more robust estimate of the temporal dynamics, we averaged the response in each condition over 100 ms time bins (similar to Bastin et al. 2013 and Vlcek et al. 2020). Then, we performed a three-way mixed ANOVA with within-subject factors **Time Bin** (ten average 100 ms time bins after stimulus onset) and **Condition** (control, egocentric and allocentric), and the between-subject factor **ROI** with the post hoc Tukey HSD test. In the Results and Discussion sections, we name the first 100 ms bin, when the BGA response to two conditions began to differ, as the ‘time of discrimination’.

## Results

### Behavioral Results

**Fig. 3:**
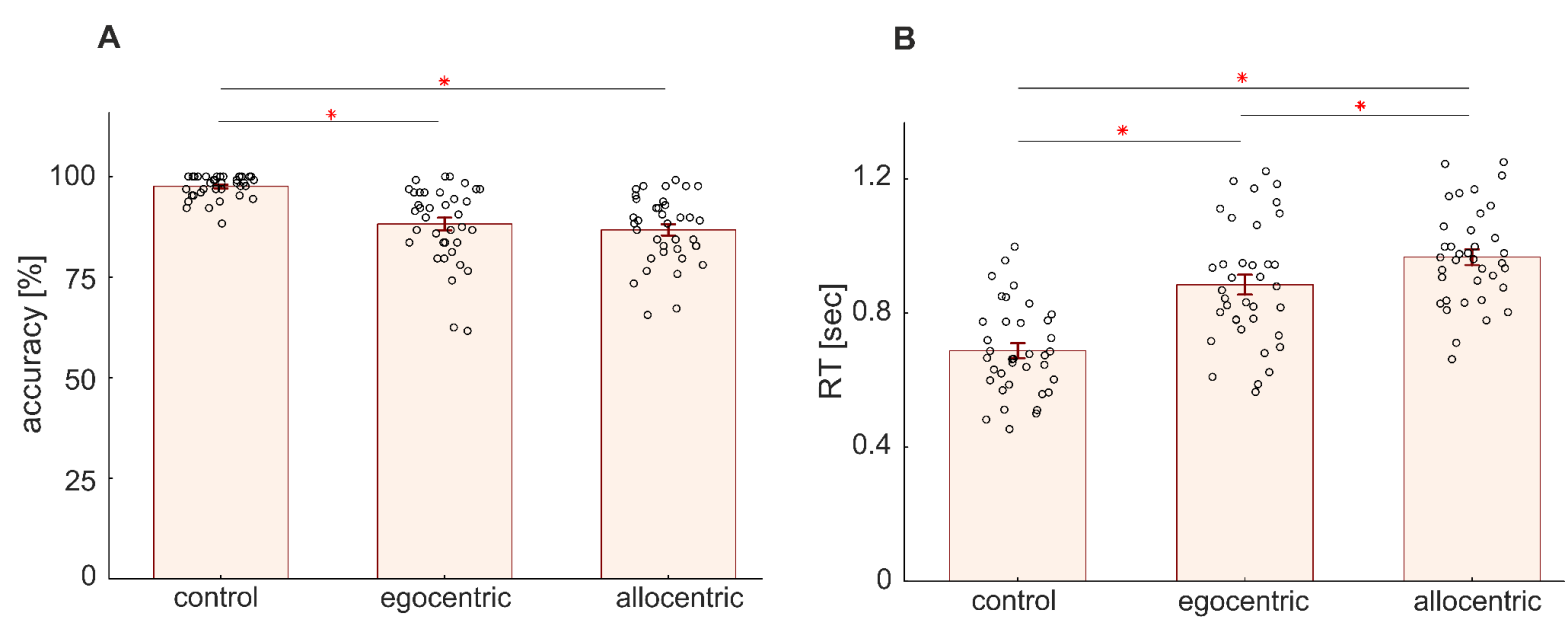
Behavioral results, accuracy **(A)**, and reaction time **(B)**, obtained from 37 patients for all three conditions (control, egocentric, and allocentric), showing significant differences between them in both measures. Each graph shows the mean and standard error of the mean; black circles represent individual data points. The red asterisk indicates a significant difference between conditions (one-way ANOVA with post hoc test, *p* < 0.05)

One-way ANOVA revealed that there was a significant effect of the **Condition** on accuracy (F(2, 72) = 42.46, *p* < 0.05) and reaction time (F(2, 72) = 313.01, *p* < 0.05) (see Fig. 3). The post hoc Tukey HSD test revealed that the patients were more successful in the control condition (here and further, results are reported in mean ± standard error of the mean form: 97.6 ± 0.5% correct) compared with both the egocentric (88.2 ± 1.6% correct, p < 0.001) and allocentric conditions (86.8 ± 1.4% correct, p < 0.001), but there was no significant difference between egocentric and allocentric conditions (p = 0.473). Reaction times significantly differed between all three conditions: patients were fastest in the control condition (688 ± 22 ms), slower in the egocentric condition (885 ± 30 ms), and slowest in the allocentric condition (967 ± 23 ms) (see Fig. 3).

### Three Categories of Task-Related Responses

We found 546 active channels showing a significant response relative to the baseline to any condition. However, the majority of these channels did not show a significant difference between conditions (382), and they probably responded to the general presentation of virtual scenes and objects. Of these 546 channels, only 164 were condition-selective (allocentric-, egocentric-, or spatial-selective). Out of these 164, nine were labeled as ‘epileptic’, either located in the seizure onset zone or manifesting strong interictal epileptiform activity. To compare the response magnitude and peak latency in epileptic and non-epileptic channels, we performed a two-way mixed ANOVA with the within-subject factor **Condition** and between-subject factor **EpiActivity**. We found no differences either in the magnitude of response (F(1, 162) = 1.37, *p* = 0.24), or in the peak latency (F(1, 162) = 2.32, *p* = 0.13). So, the epileptic activity was not related to our task. Therefore, we included these channels in further analyses. Note, however, that all epochs showing interictal spikes were excluded (see the Materials and Methods section).

**Fig. 4:**
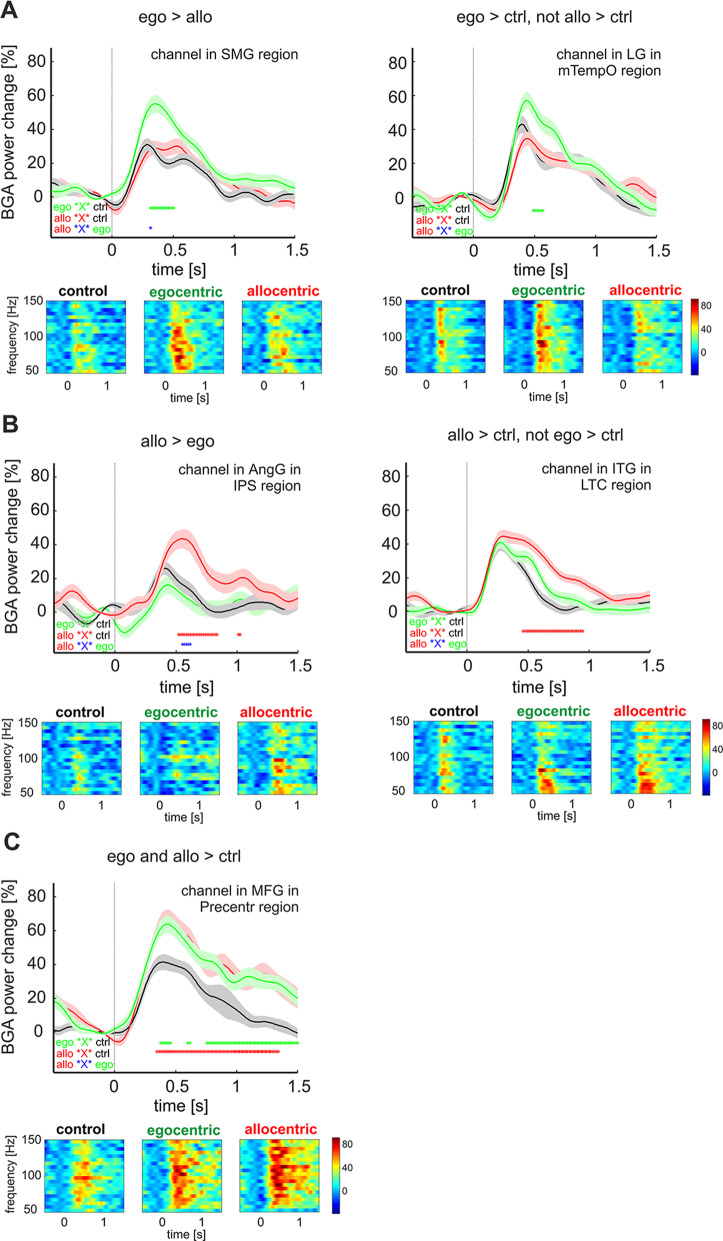
Examples of BGA responses in the individual channels, divided by category: egocentric- **(A)**, allocentric- **(B)**, and spatial-selective **(C)**. In A and B, the left column shows the response with a significant direct difference between the allocentric and egocentric conditions, while the right column shows the response with a significant difference between the respective condition and the control, but not between the control and another condition. The upper panel shows the mean ± SEM over frequency bands 50–150 Hz in the percentages of baseline activity; responses to the egocentric condition are in green, to the allocentric condition are in red, and to the control are in gray. The asterisks mark time points with the significant difference between conditions by FDR corrected Wilcoxon signed-rank at *p* < 0.05: green - between the egocentric and control, red - between the allocentric and control, and blue - between the allocentric and egocentric (both directions); the corresponding contrasts are written in the bottom left corner: ego ‘X’ ctrl, allo ‘X’ ctrl and allo ‘X’ ego, respectively. The panel below shows the BGA power responses in the frequency range of 50–150 Hz to all three conditions. Legend: SMG, supramarginal gyrus; LG, lingual gyrus; mTempO, medial temporal-occipital cortex; AngG, angular gyrus; IPS, intraparietal sulcus; ITG, inferior temporal gyrus; LTC, lateral temporal cortex; MFG, middle frontal gyrus; Precentr, precentral region

**Fig. 5:**
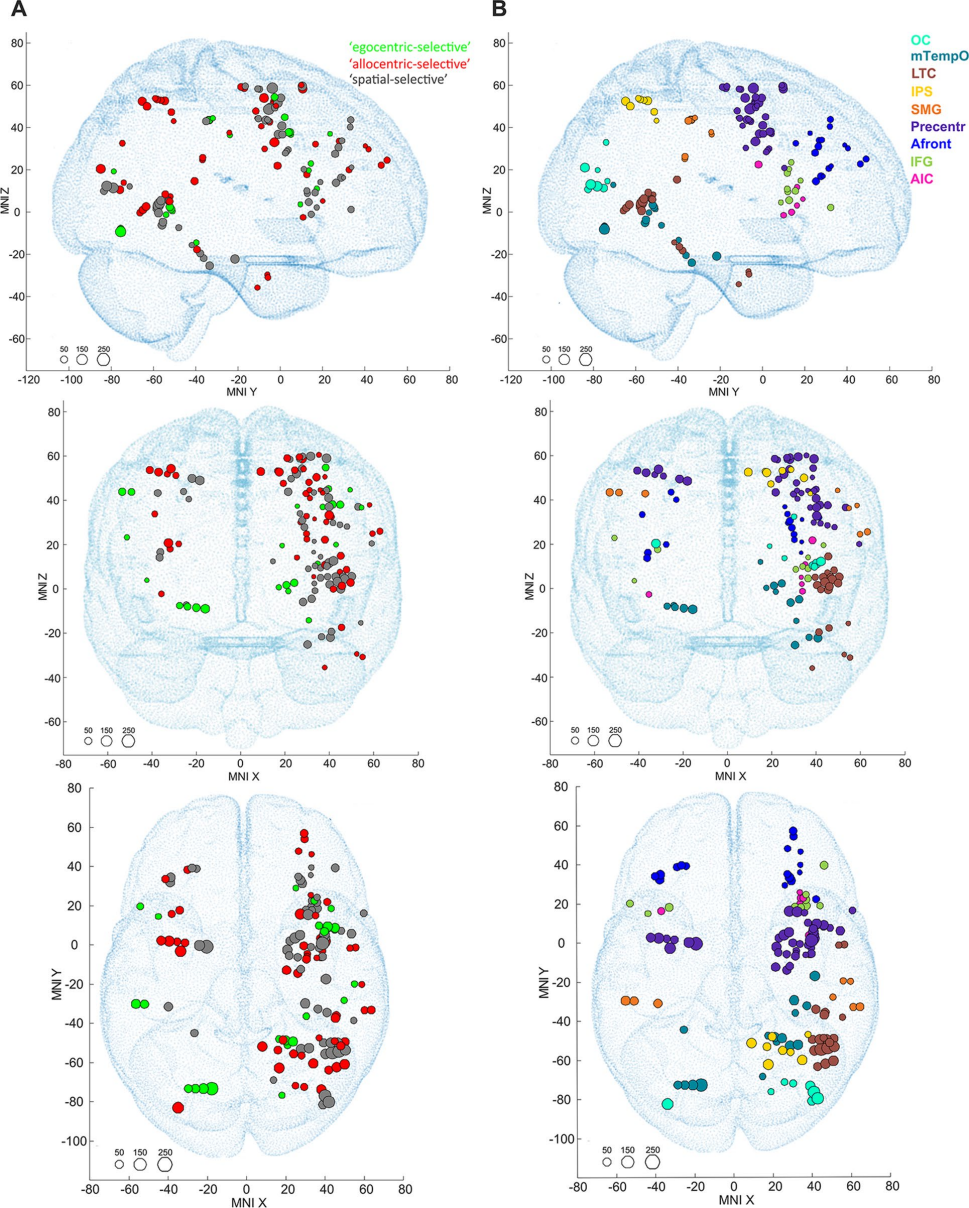
The positions of 137 channels of interest plotted in the standard MNI brain template, marked by channel category **(A)** or by ROI **(B)**. The top, middle, and bottom panels show sagittal, coronal, and axial views, respectively. The size of each point corresponds to the maximum magnitude of each channel’s response, with the scale at the bottom left in percent signal change. The adult MNI-ICBM152 head model was used as a background (Dempsey et al. 2015; http://www.ucl.ac.uk/dot-hub). Legend: OC, occipital cortex; mTempO, medial temporal-occipital cortex; LTC, lateral temporal cortex; IPS, intraparietal sulcus; SMG, supramarginal gyrus; Precentr, precentral region; Afront, anterior frontal cortex; IFG, inferior frontal gyrus; and AIC, anterior insular cortex

We grouped these 164 condition-selective channels into the brain regions described below. However, some of them were widely distributed in various brain areas where we could not record from at least three different patients and use them in the statistical analysis. Individual channels in the hippocampus (1 allocentric-selective and 1 egocentric-selective), entorhinal cortex (1 allocentric-selective), temporal pole (5 allocentric-selective), posterior angular gyrus (1 egocentric-selective), precuneus (1 allocentric-selective), retrosplenial cortex (1 egocentric-selective), anterior cingulum (2 egocentric-selective and 1 allocentric-selective), frontal operculum (1 egocentric-selective), and medial superior frontal gyrus (3 egocentric-selective) were excluded (see supplementary Fig. S3). Therefore, the final pool for analysis included 137 ChOIs, used for all the subsequent analyses (Fig. 5A). Of them, 24 were egocentric-selective (4 of them with the significant contrast ego > allo, see the examples in Fig. 4A), 54 were allocentric-selective (3 of them with the significant contrast allo > ego, see the examples in Fig. 4B), while 59 were spatial-selective (see the example in Fig. 4C, and the detailed explanation about channel types in the Materials and Methods section). Overall, we obtained about twice as many allocentric-selective channels as egocentric-selective channels.

We mapped these 137 ChOIs into the following nine brain regions (ROIs) (see also Table 1; Fig. 5): **OC** – occipital cortex but without the primary visual cortex – cuneus, middle occipital gyrus, temporo-occipital transition zone; **mTempO** – medial temporal-occipital cortex – mainly the posterior parts of lingual and fusiform gyri and the lingual-parahippocampal transition area; **LTC** – lateral temporal cortex – the inferior, middle, and superior temporal gyri; **IPS** – the area near the posterior part of the intraparietal sulcus – the superior parietal lobule and angular gyrus; **SMG** - supramarginal gyrus; **Precentr** – precentral region – the posterior part of the frontal cortex, combining the precentral gyrus and the posterior parts of the middle frontal and superior frontal gyri (with MNI ‘y’ < 20); **Afront** - anterior frontal cortex, combining the anterior parts of the middle frontal and superior frontal gyri (with MNI ‘y’ > 20); **IFG** - inferior frontal gyrus: the opercular and triangular parts; **AIC** - anterior insular cortex.

**Table 1:** Characteristics of brain regions containing the 137 channels of interest

Brain region	P	N	Spatial- selective	Allocentric-selective	Egocentric-selective	Brain structures	MNI coordinates, range			
							Abs (X)	Y	Z	
OC	5	8	3(2)	4(4)	1(1)	MOG: 5, TOTZ: 2, CUN: 1	[19, 42]	[-81, -71]	[10, 33]	
mTempO	8	16	7(5)	0	9(4): 3	LG: 8, FuG: 6, LPHT: 2	[15, 40]	[-72, -18]	[-25, 12]	
LTC	6	21	10(2)	11(5): 1	0	MTG: 13, ITG: 7, STG: 1	[38, 55]	[-63, -2]	[-35, 15]	
IPS	4	8	0(0)	8(4): 1	0	AngG: 4, SPL: 4	[9, 37]	[-62, -47]	[43, 54]	
SMG	4	8	1(1)	3(2)	4(3): 1	SMG: 8	[37, 63]	[-33, -20]	[25, 45]	
Precentr	14	43	21(11)	17(10): 1	5(4)	PreG: 21, MFG: 14, SFG: 8	[18, 59]	[-15, 15]	[21, 60]	
Afront	6	17	9(4)	7(4)	1(1)	MFG: 12, SFG: 5	[23, 41]	[20, 54]	[14, 44]	
IFG	6	10	6(3)	1(1)	3(3)	IFG: 10	[31, 51]	[13, 37]	[2, 23]	
AIC	4	6	2(1)	3(3)	1(1)	INS: 6	[33, 38]	[2, 24]	[-2, 22]	

Brain region, ROI; P, number of patients; N, number of channels in each brain region in total; the spatial-selective, allocentric-selective, and egocentric-selective columns show the number of corresponding channels with the number of patients in parentheses; additional numbers in the allocentric-selective and egocentric-selective columns after the colon show the number of channels with a significant difference in the contrast allo vs. ego; the brain structures column lists anatomical labels for all channels in the brain region, with the number of channels. The last three columns show the range ([min, max]) of X, Y, and Z MNI coordinates of each brain region. Abbreviations used: OC, occipital cortex; MOG, middle occipital gyrus; TOTZ, temporo-occipital transition zone; CUN, cuneus; mTempO, medial temporal-occipital cortex; LG, lingual gyrus; FuG, fusiform gyrus; LPHT, lingual-parahippocampal transition area; LTC, lateral temporal cortex; MTG, middle temporal gyrus; ITG, inferior temporal gyrus; STG, superior temporal gyrus; IPS, intraparietal sulcus; AngG, angular gyrus; SPL, superior parietal lobule; SMG, supramarginal gyrus; Precentr, precentral region; PreG, precentral gyrus; MFG, middle frontal gyrus; SFG, superior frontal gyrus; Afront, anterior frontal cortex; IFG, inferior frontal gyrus; AIC, anterior insular cortex; and INS, insula

The selective channels were not evenly distributed across the brain regions (χ²_(16,N=137)_ = 50.89, *p* < 0.001) (Table 1). For example, the most frequent were allocentric-selective channels in the **OC** (4/8: 3 in the MOG and 1 in the CUN), **LTC** (11/21: 5 in the ITG, 5 in the MTG, and 1 in the STG), and **AIC** (3/6). The **IPS** region contained only allocentric-selective channels (8 channels in total). Furthermore, out of all the allocentric-selective channels, 3 showed significance in the contrast allo > ego: 1 in the AnG (from the **IPS**), 1 in the MTG (from the **LTC**), and 1 in the MFG (from the **Precentr).** The prevailing numbers of egocentric-selective channels were found in the **mTempO** (9/16: 7 in the LG, 1 in the LPHT and 1 in the FuG), and the **SMG** (4/8). In addition, 4 of them showed significance in the contrast ego > allo: 3 in the LG (from the **mTempO)**, and 1 in the **SMG.** The spatial-selective channels, i.e., responding to both egocentric and allocentric tasks compared to the control, were most frequent in the frontal cortex: **Precentr** (21/43), **Afront** (9/17), and **IFG** (6/10), but their notable numbers were also found in the **mTempO** (7/16), and **LTC** (10/21).

It may be questioned in simple images like the ones used in our test whether the subject used ‘true’ egocentric and allocentric strategies for estimating distances within the presented scene. Instead of forming a three-dimensional mental image containing objects in the scene and using it for the spatial decision, one could use non-spatial strategies, like ‘the larger object is closer to me’ or others (see a description of other strategies in the Materials and Methods section). To check whether the allocentric-selective channels in the **IPS**, **OC**, and **LTC** regions represent true allocentric coding and the egocentric-selective channels in the **mTempO** and **SMG** regions represent true egocentric coding, we performed an additional analysis of all the potential non-spatial strategies that patients may use (see supplementary Fig. S1 and Supplementary Results). Summarizing both behavioral (see supplementary Table S1) and iEEG results (see supplementary Table S2) of this analysis across the different images used in the test, we are able to consider the egocentric-selective activation in the **mTempO** and **SMG** to be associated with true egocentric spatial coding and the allocentric-selective activation in the **OC, LTC**, and **IPS** to be associated with true allocentric spatial coding.

### Allocentric and Egocentric Selectivity in the Brain Regions

Most of the ROIs analyzed above contained channels with all three types of selectivity (allocentric-, egocentric-, and spatial-selective channels). Therefore, in the second set of analyses, we focused on mapping different characteristics of egocentric and allocentric responses across the brain regions independently of the individual channel selectivity. We compared the response magnitude and temporal characteristics of responses to each condition, such as peak and onset latency, across the brain regions.

**Fig. 6:**
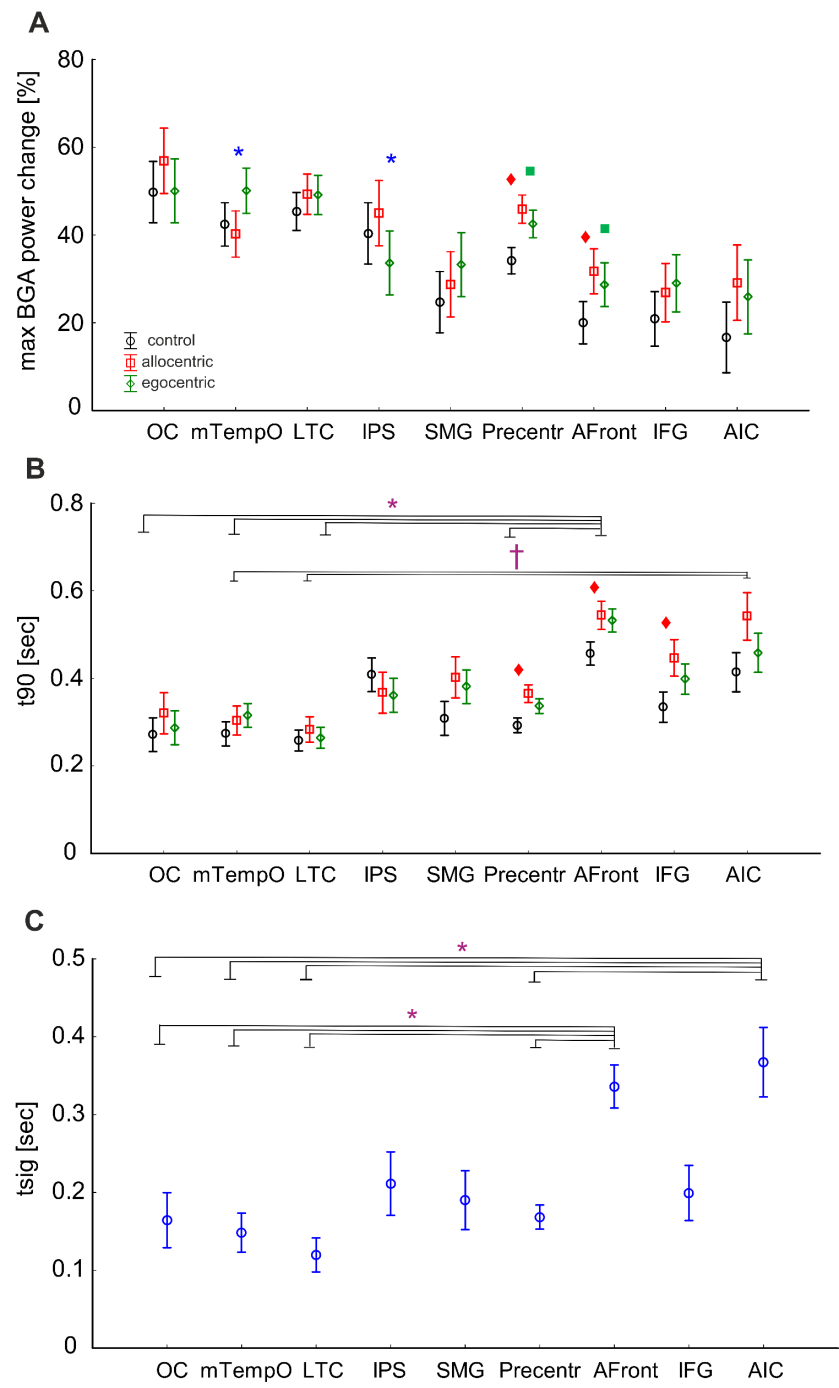
Measures of the magnitude **(A)**, peak latency **(B)**, and onset latency **(C)** of the BGA responses to individual test conditions (allocentric, egocentric, control) of all 137 channels of interest (egocentric-, allocentric-, and spatial-selective together) sorted by ROI. Plots A and B show the results of a post hoc test on two-way interaction, p < 0.05 (Condition x ROI). Plot C shows the post hoc test results on the main factor ROI, p < 0.05 (all conditions are shown together). The blue asterisk (*) reflects the difference between allocentric and egocentric conditions, the red rhombus (◊) between allocentric and control conditions, and the green square (■) between egocentric and control, within the same ROI. The violet asterisk (*) reflects the difference between ROIs for all conditions, while the violet symbol † shows the difference between ROIs only for the allocentric condition

To find differences in the BGA responses between each condition and brain region, we first examined the response magnitudes (the maximum increase of BGA in the percentage of baseline activity) (see Fig. 6A). To this end, we used two-way mixed ANOVA with the within-subject factor **Condition** and between-subject factor **ROI** with the post hoc Tukey HSD test. This analysis showed a significant effect of both main factors (factor **Condition**: F(2, 256) = 33.9, *p* < 0.01; factor **ROI**: F(8, 256) = 3.2, *p* < 0.01) and their interaction (F(16, 256) = 5.4, *p* < 0.01). After applying the post hoc test, we found differences between allocentric and egocentric responses only in two ROIs: **IPS** and **mTempO**. In the **IPS**, the average response peak was higher for the allocentric task, while in the **mTempO**, it was higher for the egocentric task (see also their time-frequency responses in 50–150 Hz in supplementary Fig. S4). In the frontal cortex (**Precentr, Afront**), the response peak was higher for both allocentric and egocentric conditions compared to the control.

Subsequently, we compared the peak latency across ROIs and conditions (see Fig. 6B). The interaction of two factors (**Condition x ROI**) was significant (F(16, 256) = 1.84, *p* < 0.05), but the post hoc test did not reveal a significant difference between egocentric and allocentric conditions in any ROI. However, in the frontal cortex, the response peak was delayed (**Precentr, Afront, IFG**) for an allocentric condition compared to the control, and there was no difference in response peak time between the egocentric and control conditions. Furthermore, the **Afront** region (all conditions) differed from almost all other regions (vs. **OC, mTempO, LTC, Precentr**): on average, the response peak was later in this brain area. In addition, the peak of the allocentric response was later in the **AIC** than in several other brain regions, such as the **mTempO** and **LTC**.

To further specify the time course of spatial coding, we also compared onset latency (the first time bin at which a significant p-value was observed relative to the baseline, tsig). A two-way mixed ANOVA showed that the interaction of two factors (**Condition x ROI**) was not significant for tsig (F(16, 234) = 0.86, p = 0.61), but the main factor **ROI** was significant (F(8, 117) = 7.42, *p* < 0.01). The post hoc test revealed that, regardless of the condition, the BGA response emerged significantly later in the **Afront** region and the **AIC** than in the **OC**, **mTempO**, **LTC**, and **Precentr** (see Fig. 6C).

### Temporal Dynamics of Egocentric and Allocentric Coding

**Fig. 7:**
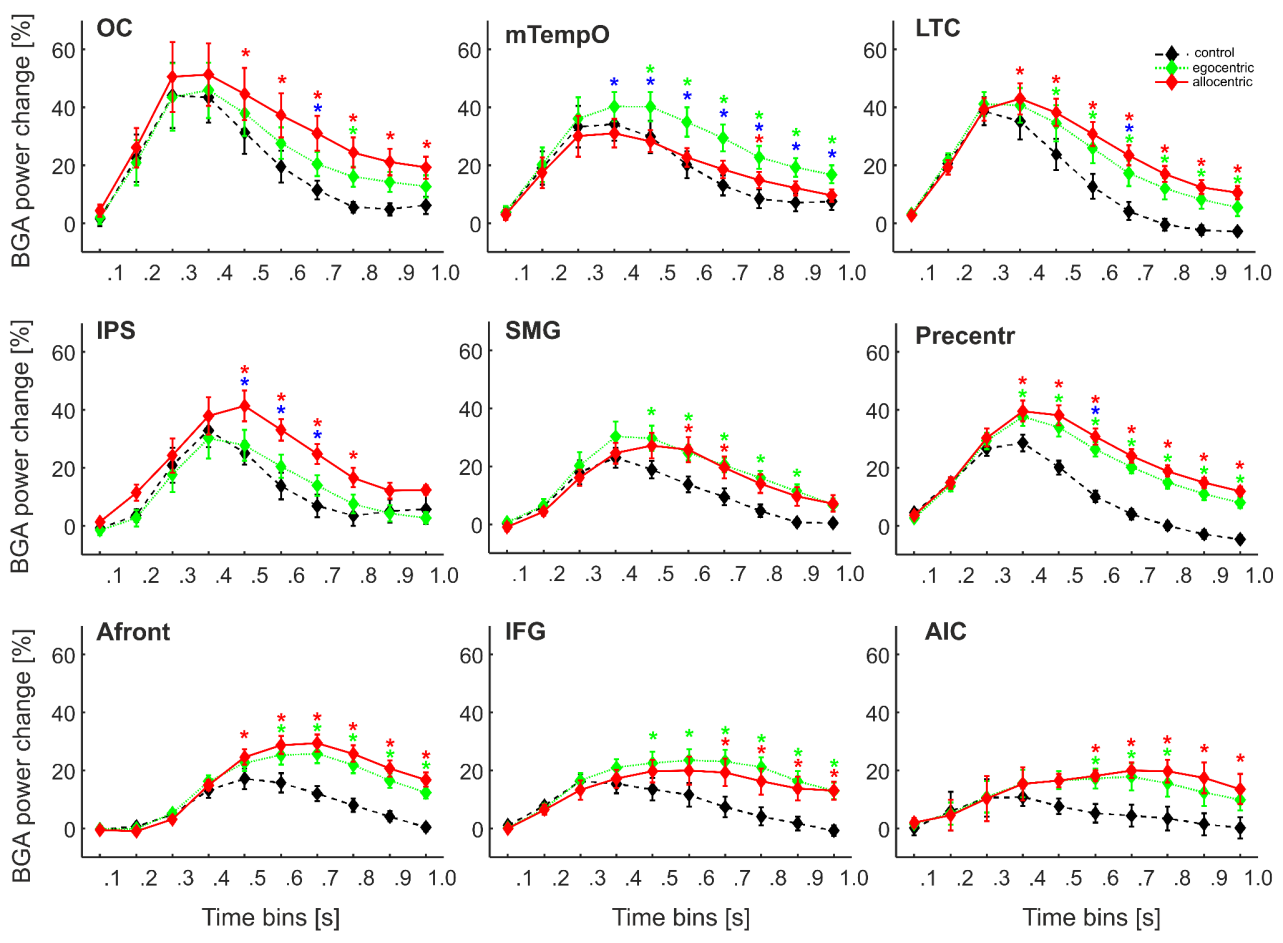
The time course of the group averaged BGA response (mean ± SEM) to individual test conditions (allocentric, egocentric, control) for all 137 channels of interest (egocentric-, allocentric-, and spatial-selective together) as a function of ROI and stimulus type. Significance markers (*) reflect the difference between the response to three conditions in each 100-ms time interval: red - between allocentric and control, green - between egocentric and control, blue - between allocentric and egocentric conditions (both directions), three-way ANOVA with post hoc test, *p* < 0.05. The x-axis labels show the upper boundary of each time interval

Next, we aimed to analyze in more detail the complete time-course of the response to the three conditions, to determine how the time course of their responses differs and develops during the whole post-stimulus period. To this end, we performed three-way mixed ANOVA with within-subjects factors **Time Bin** (ten average 100 ms time bins after stimulus onset) and **Condition** (control, egocentric and allocentric), and the between-subject factor **ROI** with the post hoc Tukey HSD test. We used such an approach to dissociate the time course of averaged BGA across all conditions in all nine ROIs (see Fig. 7). The interaction of three factors (**Condition x Time Bin x ROI**) was significant F(144, 2304) = 2.54, *p* < 0.001. The post hoc test revealed four brain regions in which the allocentric response was higher than the egocentric one at least at one 100 ms time bin: **OC, IPS, LTC**, and **Precentr**. In the **IPS**, the allocentric BGA response dissociated from the egocentric one earlier than in other regions, at the time window of 400–700 ms. Then in the **Precentr**, the response to the allocentric condition was higher than the egocentric one at 500–600 ms, although in this region, both the allocentric and egocentric responses began to differ from the control task at 300 ms after stimulus onset. Later, the allocentric response began to differ from the egocentric one in the **OC** and **LTC** at the time window of 600–700 ms.

Furthermore, post hoc test results revealed one brain region in which the egocentric response differed from the allocentric one, namely in the **mTempO** at 300–1000 ms after stimulus onset. None of the other regions showed any difference between allocentric and egocentric responses, probably due to the low number of selective channels there, but they differed in the time of discrimination from the control task. In two fronto-parietal regions, the **SMG** and **IFG**, the egocentric response began to differ from the control earlier (in both regions at 400 ms) than the allocentric response from the control (at 500 and 600 ms, respectively). In the **AIC**, both allocentric and egocentric responses began to differ from the control at 500 ms, but the duration of this difference was not the same: for the egocentric response, it ended at 800 ms, while for the allocentric one at 1000 ms.

## Discussion

Our study provides insight into the temporal dynamics of brain areas associated with allocentric and egocentric spatial RFs using iEEG. The presented analyses document several important findings.

First, our results support the view of primary egocentric and secondary allocentric representations and that allocentric representations are derived by translation of the egocentric ones during visual scene encoding (Byrne et al. 2007; Filimon 2015). In our task, both types of representations need to be constructed in each trial, as the scene configuration is variable. In agreement with this view and repeated egocentric to allocentric translations, our data documented later response peaks for allocentric responses compared to the control in all frontal regions (Precentr, Afront, IFG) and, in contrast, no later response peaks for egocentric responses in these regions. It is worth noting, however, that the difference between egocentric and allocentric response peaks did not reach statistical significance, probably due to the small number of channels and related low statistical power. Moreover, we found that the selectivity to the egocentric condition in the egocentric-selective region mTempO began earlier than the selectivity to allocentric condition in allocentric-selective brain regions such as IPS, OC and LTC. Behavioral data in our study also showed that patients performed the egocentric task faster than the allocentric one. Several behavioral studies demonstrated a similar tendency (Ruggiero et al. 2009, 2016). In a task similar to ours but using real 3D objects, Ruggiero et al. (2009, 2016) asked subjects to make egocentric and allocentric spatial judgments of the distance between objects presented on a desk. The participants made egocentric judgments (such as ‘Which object was closest to you?’) much faster than allocentric ones (such as ‘Which object was closest to the Cube?’). The authors also interpreted such a result that egocentric coding is primary and occurs almost automatically, while in the allocentric RF, spatial information is encoded effortfully, and additional attentional resources are required.

Second, our results broaden the dominant view of the dorsal and ventral streams supporting the egocentric and allocentric space coding, respectively (Goodale et al. 2004; Committeri et al. 2004; Zaehle et al. 2007). Despite the large overlap in allocentric and egocentric selectivity, we identified several brain areas preferably responsive to the allocentric or egocentric conditions. A high number of allocentric-selective channels was found in the IPS in the parietal cortex, and the maximum number of egocentric-selective channels was found in the mTempO region consisting mainly of lingual and fusiform gyri. Our data complement several other studies, suggesting the role of the medial temporal cortex in egocentric and parietal cortex in allocentric coding, as discussed below. In addition, besides the large overlap, we found a higher number of allocentric-selective regions than egocentric-selective ones, and also more allocentric-selective than egocentric-selective channels. These proportions agree with the view that allocentric coding is supported by most of the egocentric-related regions but with additional brain areas involved. A similar tendency was also observed in several fMRI studies (Committeri et al. 2004; Zaehle et al. 2007) and a recent meta-analysis (Li et al. 2021) showing less activation in the egocentric compared to the allocentric task.

### Temporal Processing of Allocentric and Egocentric Spatial Information

In agreement with our hypothesis, we observed differences in the temporal processing scheme for allocentric and egocentric RFs, specifically in the brain regions with a major proportion of channels responding to both RFs. By showing an earlier temporal profile of the egocentric response compared to the allocentric one, these findings favor the hypothesis that egocentric spatial coding is the primary process, and allocentric representations are derived from egocentric transformations (Filimon 2015; Ruggiero et al. 2009). Our data document these different temporal profiles in both response peak latency and 100 ms time bins analysis. In the frontal regions (Precentr, IFG, Afront), the allocentric condition showed a later response peak than the control. By contrast, the egocentric condition did not differ in the response peak time from the control. In addition, the egocentric response in the IFG and SMG regions began to differ significantly from the control earlier than the allocentric responses (at 400 and 600 ms after stimulus onset, respectively). The difference in peak latency may potentially be affected by the difference in the reaction time of behavioral responses, as the participants were faster in control than in the allocentric condition. However, we discarded from our analysis all late responses with the BGA peak after 800 ms and those apparently aligned to the key press time on the plot of all individual epochs. Therefore, we suppose that these EEG responses were not related to the movement but rather to the stimulus and spatial RF processing.

Importantly, our findings suggest an interaction between the three allocentric-selective brain regions, with at least three processing stages. In the first stage, the OC region responds similarly to all three conditions at about 300 ms after the stimulus onset. The location of the task-responsive channels in the OC region corresponds to the MNI coordinates of the scene-selective occipital place area (OPA) (Dilks et al. 2013; Nakamura et al. 2000). Furthermore, its response time is similar to the time of discrimination of scenes from objects observed in our previous iEEG study (242 ms, Vlcek et al. 2020) in the OPA and to the latency of scene presentation in a magnetoencephalography study (MEG) in a region close to the OPA (300 ms, Sato et al. 1999). Thus, the first processing stage might be involved in encoding general spatial layout information in our task. The second processing stage was observed in the IPS at 400–700 ms after stimulus onset, where the difference between allocentric and egocentric responses first emerged, with larger allocentric ones. In contrast, selectivity to the allocentric condition in the OC region appeared rather late, at 600–700 ms after stimulus onset; similar latency of the allocentric-selective response was also found in the LTC, the third region with a higher proportion of allocentric-selective channels. The IPS is a part of the dorsal attentional network (DAN) and has been linked to attentional selection (Ptak 2012). The IPS might send top-down attentional modulations to the OPA and LTC, triggering a BGA increase at the third processing stage, although additional studies involving functional connectivity methods are required to test this hypothesis. For OPA, this top-down effect may be represented in guiding participants’ attention to the position of the yellow mark located on the border between the floor and walls. The OPA seems to represent environmental boundaries regardless of their configuration (Kamps et al. 2016; Julian et al. 2016). In our task, the participants could use the distance from the arena wall to make allocentric judgments, a strategy accompanied by BGA responses in the OPA.

Furthermore, our data indicate early egocentric processing in the ventral visual stream. The mTempO was the region with the maximal proportion of egocentric-selective channels and the only one with a phase of egocentric response selectivity, as discussed in the next section. The average egocentric BGA response here was higher than the allocentric one at 300–1000 ms after stimulus onset. This finding is unique, as none of the previous electrophysiological studies focusing on egocentric coding reported any specific timing of this information processing (Bastin et al. 2013; Kunz et al. 2021). Bastin and his colleagues (2013) described a common phase for allocentric and egocentric processing at 400–600 ms after stimulus onset in the PPA, located more anteriorly than the mTempO. Notably, in our study, the selectivity to the egocentric response in the mTempO began earlier (at 300 ms) than the selectivity to allocentric response in allocentric-selective brain regions (IPS at 400 ms, OC and LTC at 600 ms). Although in the ventral stream (see Byrne et al. 2007), this difference seems to favor the hypothesis that egocentric spatial coding is a more primary process relative to allocentric coding (Filimon 2015).

In the dorsolateral prefrontal cortex (the Afront region), the onset latency of the BGA response and its peak for all conditions was delayed (by about 150 ms) compared to almost all other regions. At later stages of information processing, the dorsolateral prefrontal cortex may coordinate and integrate the functioning of other brain regions involved in spatial processing (Tanji and Hoshi 2008).

### Cortical Regions Selective for Egocentric and Allocentric Spatial Coding

Our results broaden the dominant view of egocentric and allocentric coding being associated with the dorsal and ventral visual streams, respectively (Goodale et al. 2004). Regarding the egocentric selectivity, besides several egocentric-selective channels in the SMG, we found their maximal proportion in the ventral stream, in the mTempO region consisting mainly of lingual and posterior fusiform gyri. It seems unexpected to observe activity associated with the egocentric RF in the ventral occipito-temporal cortex (ventral visual stream) as the opposite results for the allocentric task were found in several fMRI studies (Committeri et al. 2004; Galati et al. 2000), although more anteriorly (including PPA). However, our finding of egocentric-selective channels in this region is supported by several facts. Firstly, we cannot consider activity observed in mTempO artefactual as responses in these channels were obtained from four patients. Secondly, three channels in this region, in the posterior lingual gyrus, were more selective than most egocentric-selective channels showing a significantly higher response magnitude for the egocentric condition than for the allocentric one. There were only four channels with these characteristics in total. Thirdly, two of the four patients having egocentric-selective channels in the mTempO most likely used a true egocentric strategy rather than other strategies, e.g., based on apparent object-size features. In the supplementary analysis, we found no differences in their behavioral response accuracy between any image type in the egocentric condition. Similarly, in at least two egocentric-selective channels in the mTempO region obtained from two patients, we did not find differences in the magnitude of BGA across all image types. All these data seem to confirm the involvement of the mTempO region in egocentric spatial coding in our task.

The involvement of the medial temporal-occipital cortex in egocentric representations was shown in several lesion studies (Nyffeler et al. 2005; Weniger and Irle 2006). In a single case study in a patient with the destruction of the parahippocampal, fusiform, lingual, and medial occipito-temporal gyri, and using a paradigm of memory-guided saccades, apart from an obvious allocentric deficit, Nyffeler et al. (2005) detected an additional impairment of the egocentric coordinate frame. The role of the parahippocampal cortex was also documented in studies using a virtual maze without any landmarks where participants presumably used an egocentric navigation strategy. During navigation in this maze, the parahippocampal and lingual gyri were active (Weniger et al. 2010), and patients with lesions to the right posterior parahippocampal gyrus had difficulties finding the goal (Weniger and Irle 2006). This parahippocampal activation may be potentially associated primarily with analyzing the appearance of important navigation decision points (Janzen and van Turennout 2004). In addition, a recent paper using single-cell recordings in epileptic patients identified egocentric bearing cells in the medial temporal lobe with their highest proportion in the parahippocampal cortex (Kunz et al. 2021). These neurons were discovered during a virtual navigation spatial memory task and are considered to encode egocentric directions and distances from the observer toward reference points in space and, therefore, may represent the basis for egocentric representations. Finally, in a scalp EEG study using sparse augmented reality mazes where healthy participants navigated while being blindfolded, the lingual gyrus theta EEG power decreased across trials during spatial learning (Miyakoshi et al. 2021). According to the authors, this theta power reduction may represent a shift from the initial use of egocentric representations of the maze built on local proprioceptive and sensory feedback signals to the use of allocentric map-like representations. Notably, MNI coordinates of the cluster showing a decrease in theta band overlapped with egocentric-selective channels in the posterior lingual gyrus in our iEEG study.

Regarding the allocentric selectivity, besides allocentric-selective channels observed in the ventral stream areas such as OC and LTC, we found their maximal proportion in the IPS in the parietal cortex. This region, on a boundary between the angular gyrus and superior parietal lobule, contained only allocentric-selective channels, including one channel showing a significant difference in the contrast allo > ego. Furthermore, the response magnitude was higher for an allocentric than for an egocentric condition only in the IPS. Surprisingly, we did not find any egocentric-selective channels in the IPS, contrasting with the results of other studies (Ruotolo et al. 2019; Chechlacz et al. 2010).

Our finding of allocentric-selective channels in IPS is supported by several facts. Firstly, we are able to exclude the possibility of observing these allocentric-selective channels as an individual specific-finding, as we obtained eight such channels from four different patients. Secondly, our supplementary analysis suggests that patients with allocentric-selective channels in the IPS used a true allocentric strategy rather than other strategies. We found that two patients with channels in the IPS showed no difference in behavioral response accuracy between image types supporting different non-spatial strategies. In addition, in the IPS allocentric-selective channels, the iEEG data showed greater activation for the allocentric than for the egocentric condition in almost all image types, except when the three-dimensional and two-dimensional distance did not correspond to each other (Allo 3D ≠ 2D, i.e., the ball that was closer to the yellow mark in the three-dimensional space was more distant from it in the two-dimensional coordinates of the screen). This exception may suggest IPS involvement only when the subjects use a two-dimensional strategy. It contrasts, however, with fMRI studies showing a critical role in the integration of multiple depth cues and, therefore, in a representation of the 3D surface geometry of objects of posterior portions of the IPS (Grefkes and Fink 2005; Tsutsui et al. 2005). The lack of any difference in Allo 3D ≠ 2D trial types may probably be explained by low statistical power, as the subset of Allo 3D ≠ 2D images included a very small number of epochs.

Moreover, several other studies indicate that processes related to object-centered RFs may occur in the parietal cortex. In an fMRI experiment with the verbal description of spatial relations between objects, Zaehle and his colleagues (2007) found that parts of the inferior and superior parietal lobules expressed greater activation for the allocentric compared to the egocentric task. Significant responses in the right IPS unique to allocentric spatial coding (for the contrast allo > control, but not for ego > control, similar to our results) were also documented in a more recent functional near-infrared spectroscopy study (fNIRS) during a two-dimensional spatial discrimination task (Derbie et al. 2021b). According to the hypothesis that allocentric object-centered RFs are derived via mental transformations of primary egocentric RFs (Filimon 2015), IPS activation may be associated with additional attentional resources required to mentally shift egocentric spatial locations of each object into new, mentally transformed, object-relative positions. In an fMRI study explicitly focused on attentional orienting, the IPS showed the most pronounced increase of activity associated with object-centered relative to the viewer-centered RF (Wilson et al. 2005).

In our iEEG study, we did not find any allocentric-selective channels in the PPA and medial occipitotemporal cortex more generally, although an fMRI (Committeri et al. 2004) and another follow-up iEEG study (Bastin et al. 2013) showed posterior parahippocampal involvement during the allocentric task. Possibly, medial occipitotemporal cortex, including PPA, is only involved in the world-centered allocentric RF use and may reflect the coding of the current spatial relationships between the viewer and the whole environmental geometry (see reviews Galati et al. 2010 and Moraresku and Vlcek 2020). In contrast, the LTC seems to be associated with object-centered RF, as our experimental design included the object-centered allocentric condition in which the subjects did not need to focus on the whole environmental geometry but rather on the local spatial relationships between objects. Our finding of BGA responses to the allocentric condition in the LTC is consistent with the results of several fMRI studies (Committeri et al. 2004; Saj et al. 2014; Zaehle et al. 2007). For instance, Committeri et al. (2004) observed greater activation in the bilateral lateral occipitotemporal cortex, including inferior temporal and occipital gyri, in the contrast object-centered relative to viewer-centered condition, i.e., when participants judged which of two objects was closer to the target object ignoring the surrounding environment.

To sum up, our data, together with other recent studies, suggest that the medial temporal-occipital cortex could also be involved in egocentric coding of space and the parietal cortex – in allocentric coding.

### Study Limitations

General complication with human iEEG is the limited coverage of the brain. Although we recorded data from 4586 bipolar channels in 37 patients, some brain areas were still covered sparsely, such as the posterior parts of the occipital and the parietal cortex. Furthermore, the right hemisphere was covered quite densely (72% of all recording sites) as opposed to the left hemisphere. Because of the unequal distribution of selective channels in both hemispheres, we grouped and analyzed them together that limited us in finding any laterality in BGA responses.

The iEEG data may reflect the abnormal brain activity of epileptic patients. However, we excluded all trials showing interictal epileptiform discharges, and channels labeled ‘epileptic’ differed from non-epileptic channels neither in the magnitude of response nor in the peak latency. Moreover, for each brain region analyzed, we required iEEG activations from at least three different patients. Therefore, we are convinced that our data reflect primarily physiological mechanisms.

The low proportion of selective channels from all that were implanted is noteworthy. Of the 4586 implanted channels, 546 were responsive in our task (11.9%). This proportion is similar to other iEEG studies (Vidal et al. 2010; Vlcek et al. 2020), where around 17% of channels were responsive. The slightly lower proportion in our current study is probably connected to the specificity of our stimuli, which were all very similar spatial scenes. Of these responsive channels, only 164 were condition-selective (4% of all implanted), which we expected given the identical visual stimuli and conditions differing only in the type of question presented before.

To some extent, our results can be influenced by our experimental design. In the test images, we used a first-person view, which is more natural for humans and usually used during real navigation. However, in a bird-eye view, allocentric estimation of distance could be potentially easier than in a first-person view because of more visible distances that might be reflected in iEEG results.

Also, our results can be potentially affected by the subjects’ position during the experiment. While in fMRI studies, participants lie in a scanner, the iEEG study enables patients to perform the experimental task in an upright position, i.e., while sitting in bed. This difference may potentially affect neural activities, as sitting in an upright position may facilitate the translation of body coordinates for spatial coding. One MEG study has already revealed differences in neural activity between lying supine and sitting upright (Lifshitz et al. 2017). Source-localization analysis showed that sitting upright versus lying supine was associated with higher beta and gamma activity in the broad parietooccipital region and lower activity in prefrontal regions across a range of bandwidths.

## Conclusion

In our study, we documented the temporal and regional interactions between egocentric and allocentric spatial coding in the human brain. The egocentric selectivity in our egocentric-selective region, the medial temporal-occipital cortex, began earlier than the allocentric selectivity in our allocentric-selective brain regions, the intraparietal sulcus, occipital and lateral temporal cortex. In the frontal regions, allocentric responses also peaked later. Furthermore, we found a large overlap between egocentric and allocentric coding in the spatial domain, both at the level of individual bipolar channels and brain regions, as well as in the temporal domain. Still, we identified several egocentric- and allocentric-selective brain areas, which, however, were not confined to the dorsal and ventral visual streams, respectively. Overall, our findings indicated that there were more regions and channels that were selective to allocentric than egocentric reference frame, which supports the idea that allocentric coding is supported by most egocentric-related regions but with additional brain areas involved. Our results favor the hypothesis that egocentric spatial coding is more primary and occurs almost automatically, while allocentric representations are likely derived from egocentric ones and require additional attentional resources. Future studies may also address the functional connectivity between these areas and clarify the information flow involved in the egocentric and allocentric networks.

### Electronic Supplementary Material

Below is the link to the electronic supplementary material.

Supplementary Material 1

## Data Availability

The data from this study will be available on request from the corresponding authors.
