# Supplementary material for: Timing of Allocentric and Egocentric Spatial Processing in Human Intracranial EEG
Source: Brain Topogr. 2023 Jul 21;36(6):870–89. doi: 10.1007/s10548-023-00989-2 (PMC10522529; doi:10.1007/s10548-023-00989-2)
Supplement: Supplementary file 1 — Supplementary Material 1 [file 10548_2023_989_MOESM1_ESM.docx]

# Supplementary Information

#### Supplementary Results

Our results may have potentially been affected by the use of non-spatial strategies other than egocentric and allocentric ones. Instead of estimating three-dimensional scene distances, the subject may have used simple strategies employing features such as apparent object-size characteristics or two-dimensional on-screen distances. The design of our experiment included images that differed in terms of these strategies that patients may potentially use instead of spatial estimates (see more in the Materials and Methods section, Stimuli and Task sub-section, and Fig. S1). Therefore, we were able to perform a detailed analysis of behavioral and iEEG responses for all potential non-spatial strategies for patients in whom task-related BGA responses were found.

Firstly, we analyzed the behavioral response accuracy in those patients who contributed to the pool of egocentric-selective channels with predominant numbers in the **mTempO** and **SMG** (Table S1A) and of allocentric-selective channels with predominant numbers in the **OC, LTC,** and **IPS** (Table S1B). We assumed that if the patients relied more on one specific non-spatial strategy, they would make more errors in trials not in accordance with this strategy (i.e., a different type of trials). For instance, if, during the egocentric condition, patients consistently used the strategy - the larger ball is closer to me (Fig. S1C) - instead of the true egocentric strategy, they would make more errors in trials in which a smaller ball was closer. The same principle was applied to all possible pairs of trial types (Fig. S1A, B, C, and D). We expected to find no difference in behavioral accuracy between the different trial types for the same condition if patients made true egocentric and allocentric judgments. As for egocentric-selective regions, we found that in two patients having channels in the **mTempO** and two others having channels in the **SMG,** there was no difference in behavioral response accuracy between any trial type in the egocentric condition (Table S1A, rows with only n.s.). In the allocentric condition, three patients with channels in the **OC**, two patients with channels in the **IPS**, and five patients with channels in the **LTC** had no difference in behavioral response accuracy between any trial type (Table S1B, rows with only n.s.). Therefore, based only on the behavioral results, we found that at least two patients having selective channels in each egocentric- or allocentric-selective brain region presumably used true egocentric and allocentric strategies.

The next step was to check how the iEEG activity is preserved in egocentric- and allocentric-selective brain regions across different trial types. We assumed that if we find egocentric-selective channels in all trials together to be egocentric-selective also in all pairs of specific trial types, their activity may be associated with true egocentric coding. The brain regions containing mostly such egocentric-selective channels (e.g., the **mTempO**) may then be also associated with true egocentric coding. The same assumption was used for allocentric coding in allocentric-selective channels and the associated brain regions. To this end, we selected four pairs of trial subsets of each trial type (Fig. S1) in a series and performed statistical comparisons of the BGA response between conditions using a Wilcoxon signed-rank test, focusing only on egocentric-selective and allocentric-selective channels (the same approach as described in the Materials and Methods section) in brain regions with their maximal numbers. We found fewer selective channels (Table S2) than in all trials together, as after such division, we obtained a lower number of epochs, and the statistical power was decreased. Nevertheless, the **mTempO** region showed stable activation across all trial types in at least two egocentric-selective channels obtained from two patients (of four), which seems to confirm the involvement of this brain region in egocentric spatial coding. The brain regions, the **OC** and **LTC,** revealed more than one allocentric-selective channel obtained from one or more patients in all trial types, which supports their role in allocentric spatial coding. Two remaining brain regions, the **SMG** and **IPS**, showed activation almost in all trial types in egocentric-selective and allocentric-selective channels, respectively, except for relative size ego = 1 (the larger ball is closer to me) in the **SMG** and Allo 3D ≠ 2D in both cases. The subset of Allo 3D ≠ 2D trials included a very small number of epochs (less than 20), which may be the reason for the lack of statistical significance between the conditions. An alternative explanation is that the IPS region, for which a subset of Allo 3D ≠ 2D trials is especially relevant as only allocentric-selective channels were found here, may be involved only when allocentric distance estimation in three-dimensional and two-dimensional coordinates corresponds to each other, i.e., when subjects primarily used two-dimensional strategy. However, summarizing both behavioral and iEEG results of this analysis across different trial types, we may consider activation in the **mTempO** and **SMG** to be associated with true egocentric spatial coding and activation in the **OC, LTC,** and **IPS** to be associated with true allocentric spatial coding.

#### Supplementary Figures and Tables


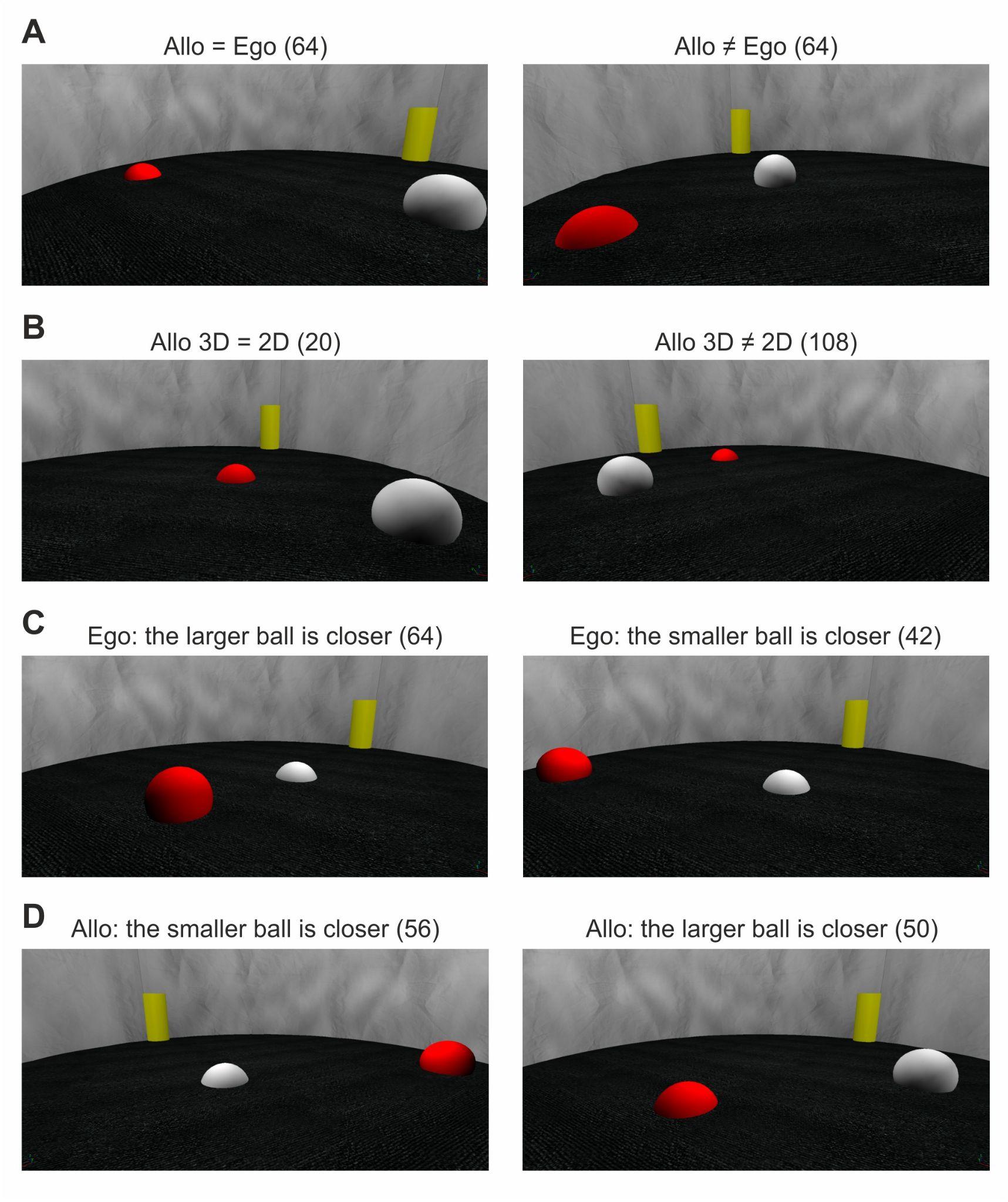


**Fig. S1** Examples of different image types used in the experimental design. In the parentheses, the number of trials is indicated for the corresponding subset of trial types. In C and D, the number of two subsets does not add up to 128, as some trials where it was uncertain which ball was larger were not included in the supplementary analysis. Note, however, that the number of trials used in the iEEG analysis may be lower due to the exclusion of epochs containing interictal epileptiform discharges or with incorrect behavioral responses

**
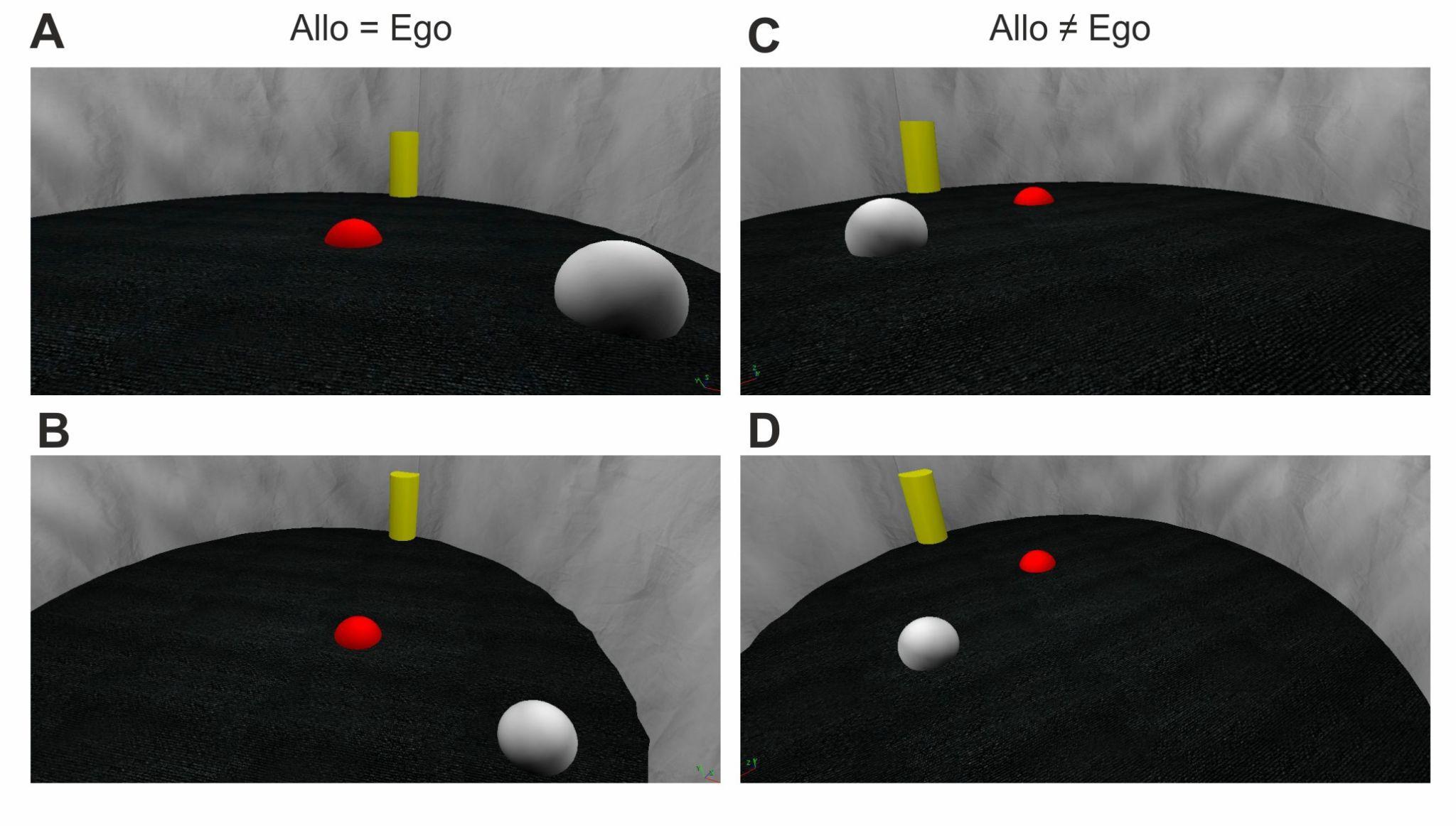
Fig. S2** Examples of images where allocentric estimation was the same in three-dimensional space and the two-dimensional coordinates of the screen (Allo 3D = 2D) (A, B), and where it was different (C, D). Images A, B, and C, D are identical, except for the viewer position. While A and C show the original images used in the test (and in Fig. S1B), where the two-dimensional on-screen distances are apparent, the overhead view images (B, D) represent better the three-dimensional distances, which was the task of the subjects to estimate

**Table S1** Behavioral results: the response accuracy across all trial types in patients with egocentric- (A) and allocentric-selective (B) channels

**A**

| ROI | Patient | Difference in response accuracy between trial types (egocentric condition only) | | | |
| --- | --- | --- | --- | --- | --- |
|  |  | Allo = Ego vs Allo ≠ Ego | Allo 3D = 2D vs Allo 3D ≠ 2D | Ego: the larger vs smaller ball is closer | Allo: the smaller vs larger ball is closer |
| mTempO | p129 | n.s. | n.s. | n.s. | n.s. |
| mTempO | p130 | * | n.s. | * | n.s. |
| mTempO | p170 | n.s. | n.s. | n.s. | n.s. |
| mTempO | p1967470 | * | n.s. | n.s. | n.s. |
| SMG | p153 | n.s. | n.s. | n.s. | n.s. |
| SMG | p160 | * | n.s. | n.s. | n.s. |
| SMG | p190 | n.s. | n.s. | n.s. | n.s. |

**B**

| ROI | Patient | Difference in response accuracy between trial types (allocentric condition only) | | | | |
| --- | --- | --- | --- | --- | --- | --- |
|  |  | Allo = Ego vs Allo ≠ Ego | Allo 3D = 2D vs Allo 3D ≠ 2D | Ego: the larger vs smaller ball is closer | Allo: the smaller vs larger ball is closer | |
| IPS, OC | VT53 | n.s. | n.s. | n.s. | | n.s. |
| IPS, OC | p160 | n.s. | n.s. | n.s. | | n.s. |
| IPS | p1670379 | n.s. | * | n.s. | | n.s. |
| IPS | p90 | n.s. | n.s. | * | | n.s. |
| OC | p132 | * | * | n.s. | | n.s. |
| OC, LTC | p129VT40 | n.s. | n.s. | n.s. | | n.s. |
| LTC | p170 | n.s. | n.s. | n.s. | | n.s. |
| LTC | p1855440 | n.s. | n.s. | n.s. | | n.s. |
| LTC | p193 | n.s. | n.s. | n.s. | | n.s. |
| LTC | p209 | n.s. | n.s. | n.s. | | n.s. |

*Asterisk (*) marks significant differences (t-test, p < 0.05, performed across response accuracy values in individual trials of each non-spatial strategy) in response accuracy for the respective patient (in rows) and the contrast of the two non-spatial strategies (in columns)*

**Table S2** BGA response selectivity across all trial types

| **ROI** |  | **All trials** | **Trial types** | | | | | | | |
| --- | --- | --- | --- | --- | --- | --- | --- | --- | --- | --- |
|  |  |  | **Allo = Ego** | **Allo ≠ Ego** | **Allo 3D = 2D** | **Allo 3D ≠ 2D** | **relative size ego = 1** | **relative size ego = 0** | **relative size allo = 1** | **relative size allo = 0** |
| mTempO (ego) | **N ego chan** | 9 | 2(3) | 3(4) | 6(5) | 1(1) | 3(2) | 2(1) | 4(3) | 2(1) |
|  | **P** | p129 p130  p170 p1967470 | p130 (p193 p222) | p130 p170 (p132 p187 p193 p222) | p129 p130 p1967470 (p170 p193 p1967470 p222) | p130 (p170) | p130 (p170 p222) | p129 p130 (p187) | p130 (p129 p170 p1967470) | p130 p170 (p222) |
| SMG (ego) | **N ego chan** | 4 | 1(1) | 1 | 2 | 0 | 0 | 2(2) | 1(1) | 1 |
|  | **P** | p153 p160 p190 | p190 (p190) | p190 | p190 |  |  | p153 p190 (p190) | p190 (p153) | p153 |
| IPS (allo) | **N allo chan** | 8 | 5 | 2(1) | 5 | 0 | 4(2) | 2(1) | 3(1) | 2(1) |
|  | **P** | VT53 p160 p1670379p90 | p160 | p160 p90 (p90) | p160 |  | p160 p1670379  p90 (VT53 p90) | p160 (VT53) | p160 (VT53) | p160 p90 (p1670379) |
| OC (allo) | **N allo chan** | 4 | 4(7) | 1(4) | 4(4) | 0(2) | 3(6) | 1(2) | 1 | 2(2) |
|  | **P** | VT53 p129VT40 p132 p160 | VT53 p129VT40  p132 p160 (VT53 p129VT40  p132 p130) | p129VT40 (p129VT40 p1967470 p90) | VT53 p129VT40  p132 p160 (p130 p132) | (p129VT40) | VT53 p129VT40  p160 (p129VT40  p132 p1967470  p90) | p129VT40 (p129VT40) | p129VT40 | p129VT40  p132 (p129VT40) |
| LTC (allo) | **N allo chan** | 11 | 8(3) | 5(2) | 8(2) | 3(4) | 9(2) | 2(3) | 5(5) | 4 |
|  | **P** | p129VT40 p170 p1855440p193 p209 | p129VT40  p170 p209 (p129VT40  p170 p1967470) | p129VT40 p170 p1855440 p209 (p170) | p129VT40  p170 p1855440  p209 (p129VT40 p1967470) | p170 p193 (p129VT40 p170) | p129VT40  p170 p1855440  p193 p209 (p170 p1967470) | p129VT40  p170 (p170 p1967470) | p129VT40  p170 p209 (p170 p1967470) | p129VT40  p170 p1855440 |

*The ROI column lists brain regions with the prevailing number of egocentric- or allocentric-selective channels (see more about the channel types in the Materials and Methods section) found in at least three patients. The rows ‘N ego chan’ or ‘N allo chan’ show the number of the egocentric- or allocentric-selective channels found to be significantly selective in all trials and in specific trial types corresponding to Fig. S1. In parentheses, there is a number of other channels found significantly selective in the particular trial type, which were, however, not selective in all trials. The ‘P’ rows list corresponding patients in whom these channels were found*

**
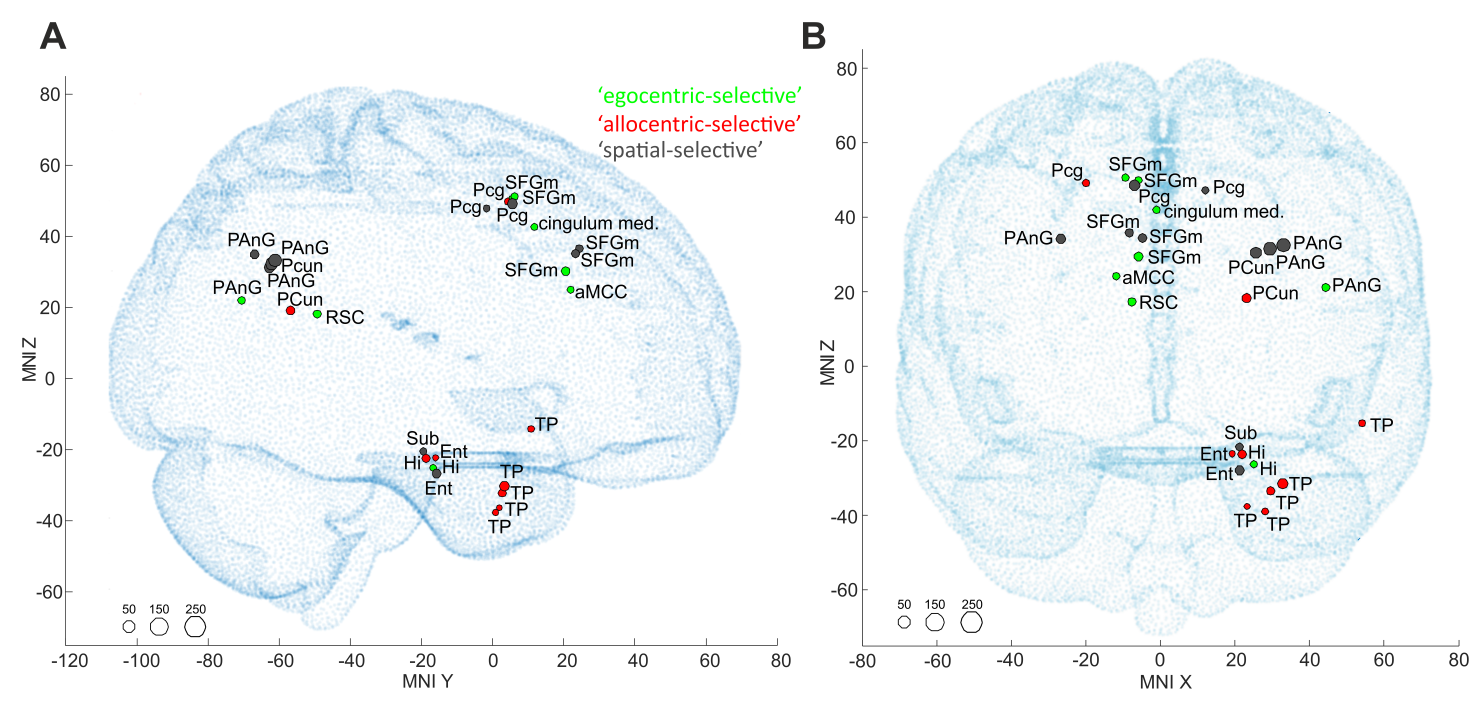
Fig. S3** The positions of the 27 channels excluded from the analysis due to their wide distribution across various brain areas recorded from less than three different patients. They are plotted in the standard MNI brain template, marked by channel category, and named with a brain structure. Panels A and B show sagittal and coronal views, respectively. The size of each point corresponds to the maximum magnitude of each channel’s response, with the scale at the bottom left in percent signal change. Legend: aMCC, anterior midcingulate cortex; cingulum med., cingulum medium; Ent, entorhinal cortex; Hi, hippocampus; PAnG, posterior angular gyrus; Pcg, paracingulate gyrus; PCun, precuneus; RSC, retrosplenial cortex; SFGm, superior frontal gyrus medial division; Sub, subiculum; TP, temporal pole

**
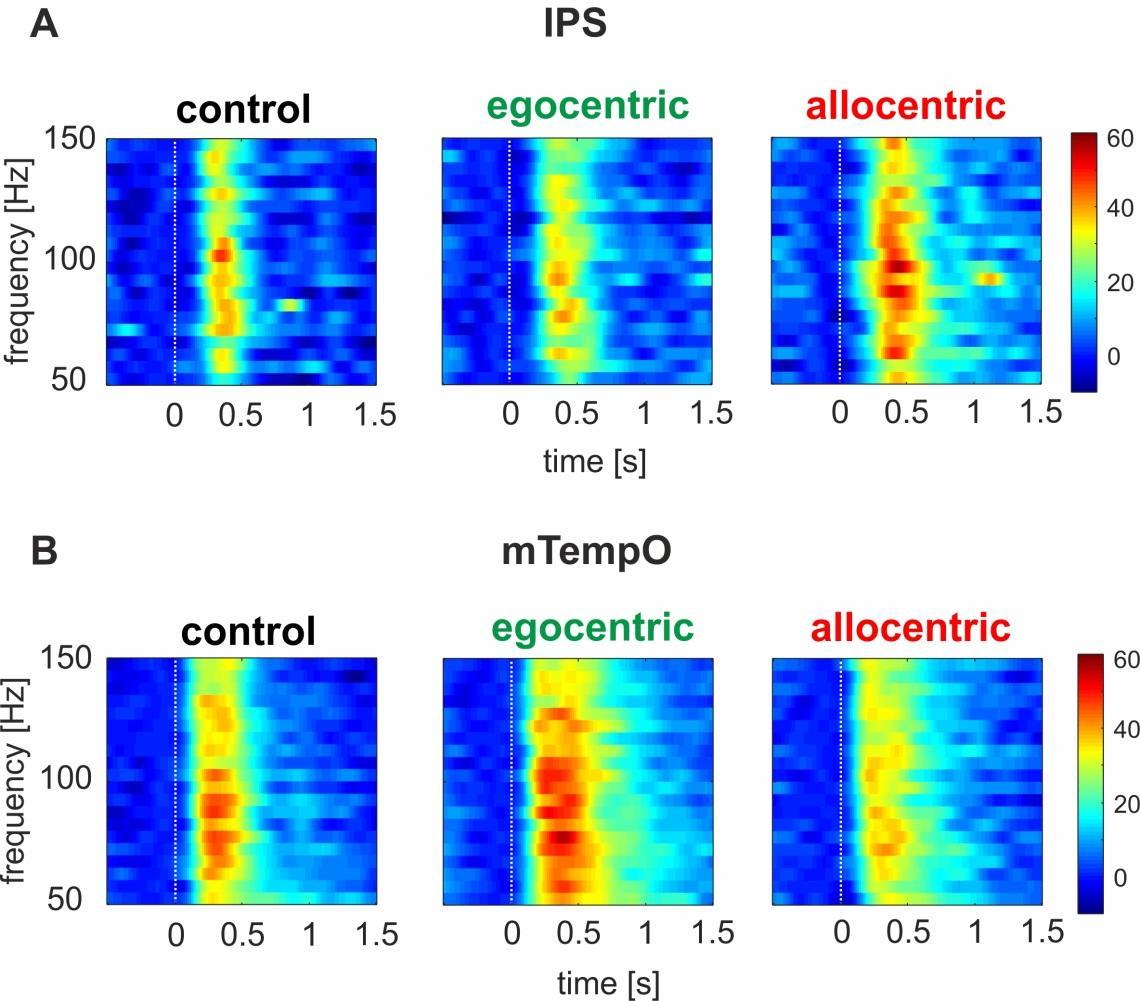
**

**Fig. S4** Broadband gamma activity (BGA) power responses to three conditions: control, egocentric, and allocentric in two selective regions - with the prevailing number of allocentric-selective channels in the IPS (A, 8 channels) and egocentric-selective channels in the mTempO (B, 16 channels): mean over channels of interest (egocentric-, allocentric-, and spatial-selective together)
